# A stroke-level large-scale database of Chinese character handwriting and the OpenHandWrite_Toolbox for handwriting research

**DOI:** 10.3758/s13428-026-03001-4

**Published:** 2026-05-11

**Authors:** Zebo Xu, Shaoyun Yu, Mark Torrance, Guido Nottbusch, Nan Zhao, Zhenguang Cai

**Affiliations:** 1 Department of Linguistics and Modern Languages, The Chinese University of Hong Kong, Shatin, Hong Kong SAR; 2 Brain and Mind Institute, The Chinese University of Hong Kong, Shatin, Hong Kong SAR; 3 Psychology Department, School of Social Sciences, Nottingham Trent University, Nottingham, UK; 4 Primary School Education, Human Science Faculty, University of Potsdam, Potsdam, Germany; 5 Department of Translation, Interpreting and Intercultural Studies, Hong Kong Baptist University, Kowloon, Hong Kong SAR

**Keywords:** Handwriting, Database, Chinese, Character, Radical, Stroke, Toolbox

## Abstract

Understanding what linguistic components (e.g., phonological, semantic, and orthographic systems) modulate Chinese handwriting at the character, radical, and stroke levels remains an important yet understudied topic. Additionally, there is a lack of comprehensive tools for capturing and batch-processing fine-grained handwriting data. To address these issues, we constructed a large-scale handwriting database in which 42 Chinese speakers each handwrote 1200 characters in a handwriting-to-dictation task. Additionally, we enhanced the existing handwriting package and provided comprehensive documentation for the upgraded OpenHandWrite_Toolbox. This toolbox allows users to easily modify the experimental design, capture the stroke-level handwriting trajectory, and batch-process handwriting measurements (e.g., latency and duration). Analysis of our large-scale database indicates that orthographic predictors affect handwriting preparation and execution at the character, radical, and stroke levels. Phonological factors influence handwriting at character and radical levels. Importantly, these lexical effects demonstrate hierarchical attenuation: they were most pronounced at the character level, followed by the radical, and weakest at the stroke level. These findings demonstrate that handwriting preparation and execution at the radical and stroke levels are closely intertwined with linguistic components. This database and toolbox offer valuable resources for future psycholinguistic and neurolinguistic research on the handwriting of characters and sub-characters across different languages.

**Supplementary Information:**

The online version contains supplementary material available at 10.3758/s13428-026-03001-4.

## Introduction

Previous research has used digital tablets to collect online handwriting data and has shown that handwriting involves multiple cognitive stages, from preparation processes, such as lexical selection and orthographic access, to execution processes that convert phonological and semantic information into orthographic output (Wang et al., 2020; Damian & Qu, 2019; Zhang & Wang, 2015). In visually complex writing systems like Chinese, these processes may operate across multiple orthographic levels, as characters consist of radicals, which further consist of smaller orthographic units such as strokes. However, most handwriting research has focused on character-level processing, leaving sub-character handwriting understudied. To address this gap, we developed a large-scale, multi-level handwriting database and upgraded an open-source toolbox for collecting and analyzing online handwriting data.

### Chinese character handwriting in the digital age

Unlike words in alphabetic scripts, Chinese characters are organized within a “square layout”, in which each character is the smallest unit that can function independently as a word with meaning (e.g., 清, *qing*_*1*_, meaning “clear”) or combine with other characters to form multi-character words (e.g., 清水, *qing*_*1*_*shui*_*3*_, meaning “clear water”). The Chinese writing system is renowned for its intricate hierarchy of orthographic levels: characters, radicals, and strokes. A character contains one or more radicals (e.g., 氵 and 青 in 清), which in turn consist of strokes (e.g., the radical 氵 consists of strokes 丶, 丶, and ⸍ ) arranged spatially within the square layout. In the character 清, which follows a left-to-right composition, 氵 appears on the left and 青 on the right. While the radical 青 (*qing*_*1*_, meaning “green”) can function independently as a character, the radical 氵 cannot. Learners must follow strict stroke order conventions when writing radicals and strokes to form characters. For instance, in 清, the radical 氵 is written first, followed by 青; within 氵, the stroke order is 丶, 丶, and ⸍. Some Chinese characters are simplex characters (独体字 in Chinese), which cannot be decomposed into smaller radicals (e.g., 年, *nian*_*2*_, meaning “year”). Although such characters may still be conventionally assigned a radical for lexicographic purposes, they lack internal radical structure in the sense described above and are written as a single integrated configuration of strokes.

Chinese characters are also known for their limited transparency between phonology and orthography, as phonemes do not map directly onto graphemes. For instance, the character 清 is pronounced *qing*_*1*_ (or /tɕʰiŋ/ in IPA), yet its phonemes (/tɕʰ/, /i/, and /ŋ/) do not correspond to specific graphemes. Although some phonetic radicals can provide pronunciation cues (e.g., 青 provides cues to the pronunciation of the character 清, *qing*_*1*_), these cues are generally unreliable: the pronunciation of a character aligns with that of its phonetic radical only about 30% of the time, according to the *Xinhua Dictionary* and primary Chinese textbooks (Shu et al., 1998; Zhou, 1978). Additionally, the pronunciation of a phonetic radical can be the same as various characters (e.g., the phonetic radical 青 and various characters 轻, 卿, and 倾 are all pronounced as “*qing*_*1*_”), and the position of a phonetic radical is not consistent (e.g., the radical 青 appears in different positions in characters like 清, 静, and 箐). This weak correspondence between phonemes and graphemes, together with the dominance of phonology-based typing systems in the digital age, has led to increasing difficulties for Chinese speakers in handwriting characters, a phenomenon known as character amnesia (提笔忘字 in Chinese; Almog, 2019; Huang et al., 2021a, b), in which individuals can recognize a character but fail to handwrite it.

### Empirical studies on Chinese character handwriting

**Fig. 1:**
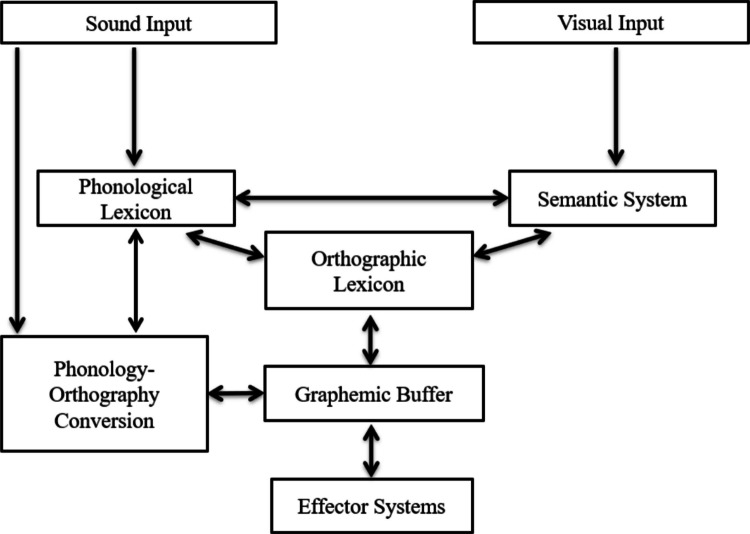
A cognitive model of handwriting, adopted from Rapp et al. (2016) and Rapp and Dufor (2011)

Chinese character handwriting involves a series of cognitive processes (see Fig. 1). In handwriting-to-dictation, for instance, when hearing a dictation phrase such as 清澈的清 (*qing*_*1*_*che*_*4*_* de qing*_*1*_, meaning the to-be-written character is 清 as in the word 清澈), handwriters first identify the phonological form of the context word *qing*_*1*_*che*_*4*_ and the target character *qing*_*1*_, along with their associated semantic representations. This information enables the retrieval of the target character’s orthographic representation from the orthographic lexicon. Evidence for the involvement of orthographic long-term memory comes from character frequency effects: high-frequency characters are written more accurately and fluently and are less susceptible to character amnesia, indicating more stable orthographic representations (Rapp et al., 2016; Rapp & Dufor, 2011; Wang et al., 2020; Lau, 2020a, b; Huang et al., 2021a, b; Xu et al., 2025a). The retrieved orthographic representations are then programmed into graphemes and stored in the orthographic working memory (the graphemic buffer; Rapp et al., 2016). The existence of a graphemic buffer in Chinese handwriting is supported by the effects of stroke number: characters with more strokes or radicals are written less accurately and/or more slowly, and are more prone to amnesia, even after controlling for frequency and other factors (Wang et al., 2020; Xu et al., 2025a; Lau, 2020a, b; Huang et al., 2021a, b). Phonology–orthography conversion also supports Chinese handwriting (Wang et al., 2020; Qu et al., 2011; Zhang & Wang, 2015): for instance, characters that are less regular (e.g., characters without a valid phonetic radical to indicate their pronunciation) are associated with lower handwriting accuracy, longer handwriting time, and higher rates of character amnesia (Wang et al., 2020; Huang et al., 2021a, b).

The cognitive model of handwriting, as illustrated in Fig. 1, has been supported by a large-scale study by Wang et al. (2020). These researchers asked 203 Chinese speakers to perform a handwriting-to-dictation task involving 200 characters randomly selected from a 1600-character pool. After including 14 lexical variables for each character, multiple regression analyses revealed higher accuracy and shorter latencies in handwriting for characters with greater regularity, higher homophone density, higher imageability, higher concreteness, higher frequency, earlier age of acquisition, fewer strokes, and greater context word familiarity. These lexical variables also influenced writing execution: shorter writing durations were associated with characters with higher frequency, earlier age of acquisition, fewer strokes, or greater context word familiarity. These results suggest that character-level handwriting makes use of both character- and sub-character-level representations: handwriters access whole-character representations via phonological information (e.g., homophone density), semantic information (e.g., imageability, concreteness), and the orthographic lexicon (e.g., character frequency). Handwriters also make use of radical-level units for phonology–orthography conversion; higher character regularity facilitates orthographic retrieval because phonetic radicals provide phonological cues that support access to orthographic representations. Finally, these stroke-level representations are maintained in the graphemic buffer before being retrieved for handwriting output (Rapp et al., 2016; Rapp & Dufor, 2011).

There have also been attempts to investigate how lexical variables influence sub-character handwriting (i.e., radical- and stroke-level writing latencies). For instance, Lau (2020b) recruited 20 native Cantonese-speaking adults to copy 211 traditional Chinese characters. Regression analyses revealed that radical writing latency (i.e., the time between completing the last radical and the pen touching the paper to begin the next radical) was longer than stroke writing latency (i.e., the time between completing the last stroke and the pen touching the paper to begin the next stroke), even after controlling for inter-stroke distance. Moreover, lower character frequency and a greater number of strokes significantly increased both radical and stroke writing latencies, with stronger effects at the radical level than at the stroke level. Lau (2020a) replicated these findings and further demonstrated that lower character regularity predicted longer radical and stroke writing latencies. These effects have been observed in adults learning Chinese as a foreign language (Zhang, 2022). The finding that character-level variables (e.g., character frequency) continually influence radical-level writing preparation (as reflected in writing latency) imply that the modulation of whole-word representations in the orthographic lexicon cascades from character-level to sub-character-level handwriting.

However, existing research on sub-character-level handwriting has mainly used a limited number of stimuli, which may limit the generalizability of findings across the full character set. This limitation is particularly problematic for Chinese, given its vast character inventory, complex structures, and variability in radical and stroke configurations. For instance, the 211 characters in Lau (2020b) represent less than 10% of the characters that literate Chinese speakers commonly encounter, potentially failing to capture the full range of orthographic, phonological, and semantic variations that exist in the Chinese writing system. The constraints of small-scale studies become even more pronounced when investigating sub-character components. With limited stimuli, it becomes difficult to disentangle the effects of various lexical variables that may be confounded at the radical and stroke levels. For instance, variables such as the number of radicals, phonetic transparency (e.g., character regularity), and the number of strokes cannot be adequately controlled in small stimulus sets. Thus, the interaction between lexical variables and different handwriting levels requires sufficient statistical power to detect.

A large-scale approach to studying sub-character handwriting would address these limitations by examining a comprehensive range of characters that better represent the diversity of the Chinese writing system. By including a substantial number of characters with varying radical compositions, stroke patterns, and lexical properties, researchers can more reliably isolate the independent contributions of different variables while controlling for potential confounds. This approach would also enable the investigation of how lexical effects cascade from the character level to radical and stroke levels across different types of characters, providing a more complete picture of the cognitive processes underlying Chinese handwriting production. Furthermore, findings from large-scale studies would have greater ecological validity and generalizability, offering insights that are more representative of real-world Chinese handwriting behavior. Indeed, recent large-scale studies have examined how character-level processing interacts with lexical effects, including phonological (e.g., character regularity and homophone density), semantic (e.g., imageability and concreteness), and orthographic factors (e.g., character frequency and number of strokes), in recognition (Sze et al., 2014; Tse et al., 2017), naming (Chang et al., 2016), and handwriting (Wang et al., 2020). This large-scale approach has also been applied to study various languages, including English (e.g., Balota et al., 2007; Keuleers et al., 2012), French (e.g., Ferrand et al., 2010), Italian (Barca et al., 2002), and Chinese (e.g., Liu et al., 2007; Wang et al., 2020).

### Tools for capturing handwriting processes

Existing handwriting research tools in general-purpose platforms can capture various measurements, such as character writing latency, character writing duration, and character pen pressure (Li-Tsang et al., 2022). However, these tools are often limited by technical constraints and researchers’ programming expertise. For instance, Wang et al. (2020) used E-Prime for Chinese handwriting experiments but could not capture online measures like handwriting time course or pen pressure. This led to less precise measurements of writing trajectories and duration (e.g., handwriting offsets were determined by keyboard presses). Specialized solutions offer more targeted functionality but come with their own limitations. The Smart Handwriting Analysis and Recognition Platform (SHARP; Li-Tsang et al., 2022) and Ductus (Guinet & Kandel, 2010) support basic experimental designs with text, picture, and audio presentations while capturing kinematic data, including writing velocity, latency, and duration. Eye and Pen (Alamargot et al., 2006) enables the synchronized collection of eye and pen movement data, facilitating research on relationships between handwriting durations and eye fixation durations (Drijbooms et al., 2020; Alamargot et al., 2010; Lambert et al., 2011; Alamargot et al., 2011). However, these specialized tools typically depend on proprietary services and dedicated hardware, limiting experimental design flexibility, since these tools offer only basic templates and require advanced programming skills for customization, thus restricting broader accessibility. Additionally, the absence of standardized protocols for recording and storing handwriting data has impeded the research community’s ability to share and compare findings across studies.

Recent handwriting programs follow the Open Science Movement, allowing researchers to use, modify, and distribute the software freely. OpenHandWrite (Simpson et al., 2021; Torrance & Conijn, 2024) is a platform designed to address the challenges mentioned above by facilitating experimental design, data preprocessing, and the sharing of handwriting research. It integrates advanced capabilities for capturing detailed handwriting trajectories, such as zero-pressure hovering movements, with the versatility of PsychoPy (Peirce et al., 2019), a widely used open-source application for building experiments. OpenHandWrite also saves and exports experimental data in standardized formats, offering an open protocol for exchanging handwriting datasets.

### The present study

As reviewed above, previous research assumes that handwriting-to-dictation involves accessing phonological and semantic information to associate with the whole-character orthographic representations from the orthographic lexicon, drawing on phonology–orthography conversion supported by radical-level information, and maintaining retrieved representations in the graphemic buffer. However, these studies either lacked precise handwriting measurements (Wang et al., 2020) or did not conduct large-scale experiments that accounted for a range of lexical variables (e.g., Xu et al., 2025a; Lau, 2020a, b). Critically, there is currently no publicly available, large-scale handwriting database that provides online measures at the character, radical, and stroke levels. The absence of such a multi-level handwriting database limits our ability to evaluate how previously proposed linguistic components modulate handwriting at different levels. To address these gaps, we collected online handwriting measures for 1200 simplified Chinese characters from 42 adult participants, yielding a comprehensive dataset that captures handwriting behavior at the character, radical, and stroke levels. Using this database, we examine how lexical variables associated with different linguistic components influence handwriting across different levels. In particular, we examined whether the effects of lexical variables follow a pattern of cascaded attenuation across the character, radical, and stroke levels: the effects of linguistic components (e.g., orthographic lexicon, graphemic buffer, or phonology–orthography conversion) cascade from character-level to sub-character-level handwriting. We also hypothesized that these components exert the strongest influence on character-level handwriting, followed by radical-level handwriting, and the weakest influence on stroke-level handwriting.

As existing tools for handwriting research are mainly proprietary products (e.g., Wang et al., 2020; Li-Tsang et al., 2022; Lau, 2020a, b), which prohibit customization for specific research requirements, another purpose of the current study was to develop a user-friendly open-access suite of tools (OpenHandWrite_Toolbox) and to use it to collect the online handwriting data in our study. Built on top of OpenHandWrite (Simpson et al., 2021), OpenHandWrite_Toolbox includes a graphical user interface (GUI) for intuitive experiment design and companion batch-processing scripts to extract critical handwriting variables, including writing latency, writing duration, and pen pressure at the character, radical, and stroke levels. The toolbox also supports annotation of fine-grained writing trajectories and the digitization of handwritten images. We aimed to provide a comprehensive and user-friendly guide to the full functionality of the toolbox, enabling a wider audience to use, adapt, and modify it for their research.

### Toolbox development

OpenHandWrite (Simpson et al., 2021) is an open-source platform that captures detailed handwriting trajectories, including zero-pressure hovering movements, integrated with PsychoPy (Peirce et al., 2019), a widely used open-source application for building experiments. OpenHandWrite saves and exports experimental data in standardized formats, offering an open protocol for sharing handwriting datasets among researchers. We developed OpenHandWrite_Toolbox on the foundation of OpenHandWrite, which includes GetWrite and MarkWrite. GetWrite supports handwriting data collection using digitizer pens and Wacom tablets within the PsychoPy environment, while MarkWrite provides visualization and markup capabilities for segmenting handwriting traces into theoretically meaningful units.

**Fig. 2:**
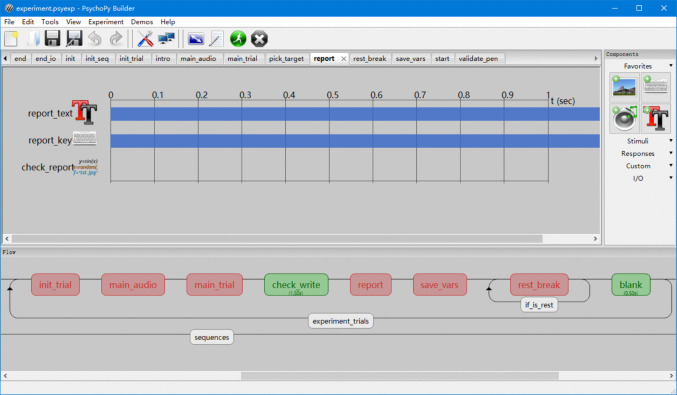
PsychoPy Builder interface. The ***lower panel*** displays the sequence of routines that define the experimental flow, with each routine represented as a ***colored box***. These routines are organized within a loop named “experiment_trials”, which controls trial iteration. The ***upper panel*** shows the contents of the selected routine (“report”), where individual elements such as the instruction text (“report_text”), keyboard response (“report_key”), and custom code (“check_report”) are arranged along a timeline. This visual interface allows users to build experiments without extensive programming

The first major component of OpenHandWrite_Toolbox is a ready-to-use experimental template that researchers can easily customize using PsychoPy Builder. While previous studies have employed GetWrite in script-based PsychoPy experiments (Fitjar et al., 2021, 2024), our template integrates its functionality directly into Builder’s graphical interface. Through Builder, researchers can visually construct experiments by connecting routines and loops. As illustrated in our experiment (Fig. 2), a typical trial comprises several routines representing stages such as audio prompt presentation, handwriting recording, and self-report collection. Once an experiment is set up using the experimental template, it can automatically record handwriting trajectories for each trial. A detailed guide to building experiments is provided in Appendix A.

**Fig. 3:**
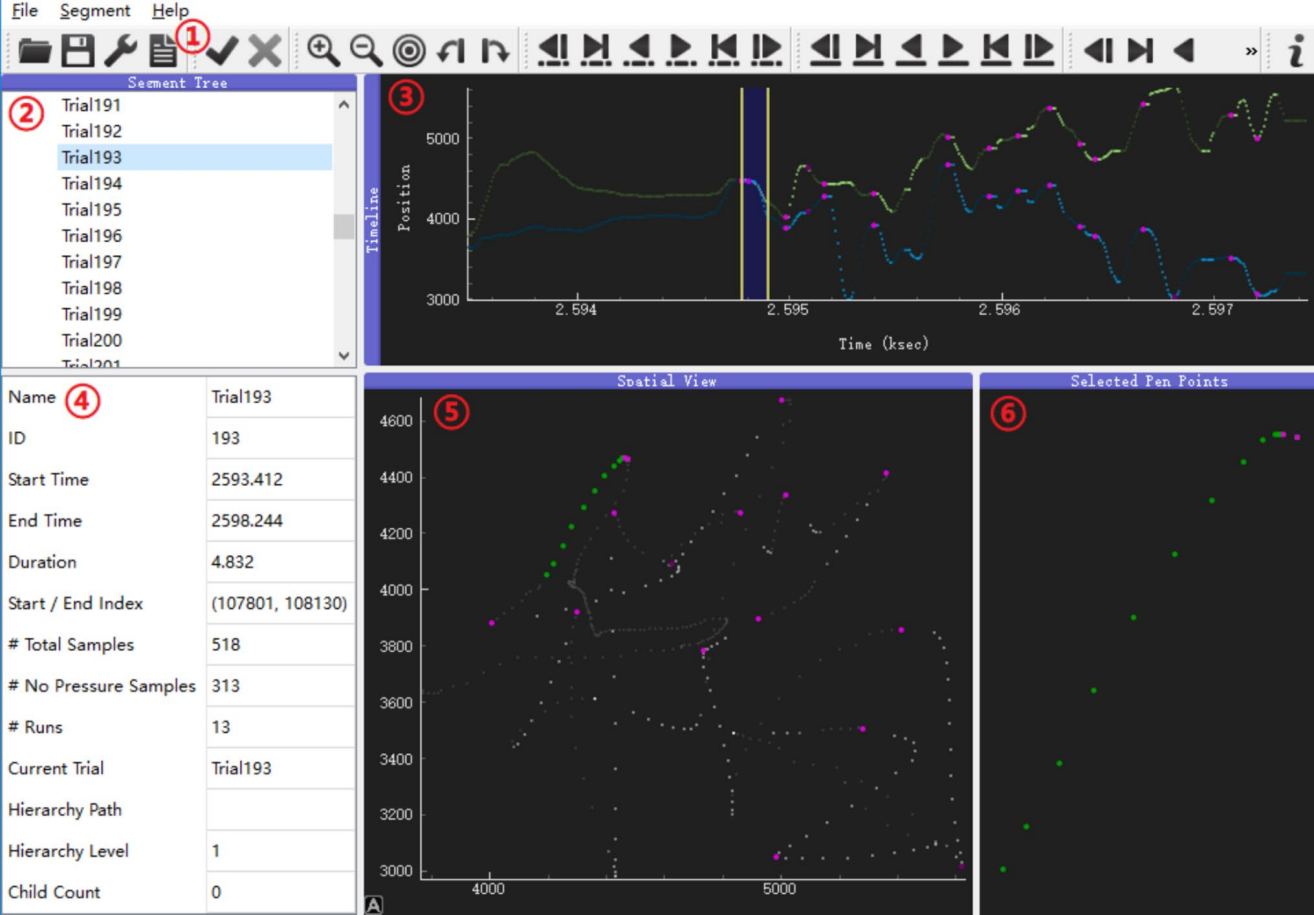
Markwrite interface. *1.* Menu bar. *2.* Segment tree, which lists experimental trials. *3.* Timeline, which displays pen points as a function of time (in milliseconds) and position (***green points*** for horizontal positions and ***blue points*** for vertical positions). *4.* Details of the pen points selected in the timeline. *5.* Spatial view, which displays the spatial view of the pen points, with the *green dots* representing the selected data point in the timeline, *purple dots* representing the onset of a stroke, and *gray dots* representing the pen in the air. *6.* Selected points, which are a zoomed-in view of the data selected in the timeline

**Fig. 4:**
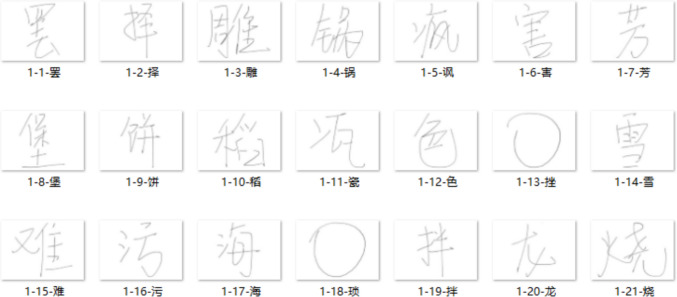
Examples of handwritten images. For the file name of each image, the first number stands for the participant ID, the second number represents item number, followed by the character name

**Fig. 5:**
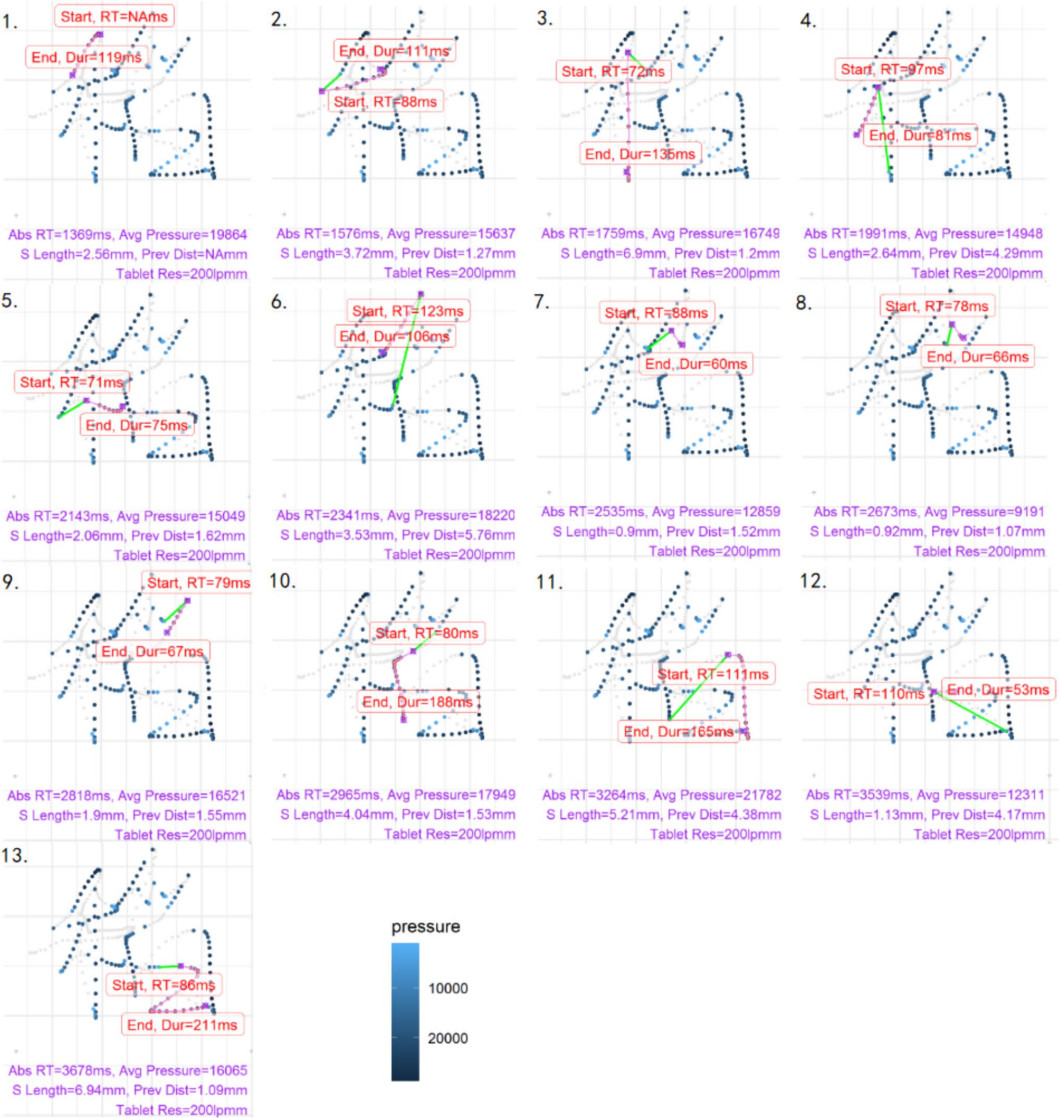
A plot showing the production for the handwritten character “稻” by one participant, with one panel for each of the 13 strokes, in the order in which the strokes were produced. The current stroke is represented by the *purple line*. The *grey dots* represent the pen-in-air movement. “Abs RT” refers to the duration from the offset of the auditory stimulus to the start of the current stroke; “Avg Pressure” refers to the average pen pressure for the current stroke; “S length” refers to the stroke length; “Prev Dist” refers to the distance between the start point of the current stroke and the previous stroke’s end point, indicated by the green line. “Start, RT” refers to the stroke writing latency. “End, Dur” refers to the stroke writing duration

The second key component of our package is a set of scripts designed to automatically compute and export handwriting metrics for analysis. We store handwriting data in the standard OpenHandWrite format, comprising timestamps, *x*- and *y*-coordinates, pen pressure, and other experimental variables. This format ensures compatibility with MarkWrite, the data visualization and segmentation tool provided by OpenHandWrite, allowing researchers to inspect and group pen samples intuitively (Fig. 3). For instance, multiple strokes can be grouped into radicals, which are essential components of Chinese characters. To extract meaningful features from raw data, our scripts automatically compute various metrics, including writing latency, duration, pen-trace length, and average pressure. These metrics can be extracted at stroke, radical, and character levels (Table 1), supporting hierarchical analysis of handwriting production. The scripts also generate character images (Fig. 4) and provide sophisticated visualizations that decompose characters into individual strokes, annotating each with associated metrics (Fig. 5). Appendix B presents a detailed walkthrough of the scripts and metrics.

**Table 1:** Description of the character-, radical-, and stroke-level handwriting metrics

Handwriting metrics	Description
Character writing latency	Duration from the offset of the stimulus to the pen tip first touching the tablet on this trial
Character writing duration	Duration from the onset of pen-tip firstly touched the tablet in this trial to the last pen sample with non-zero pressure
Character writing length	The total trajectory length of a written character is computed as the sum of distances between consecutive pen-sample points along all strokes. For each stroke, writing length was calculated by summing the linear distances between successive coordinates (*x*, *y*) recorded during pen movement; character writing length was then obtained by summing these stroke-level lengths
Character average pen pressure	Mean pressure of all stroke samples of the character
Radical writing latency	Duration between the offset of the last radical and onset of the current radical
Radical writing duration	Duration between the onset of the current radical and the end of the current radical
Radical writing length	Sum of the lengths of the strokes belonging to the radical
Radical average pen pressure	Mean pressure of the radical’s pen samples
Radical distance	Linear distance between the first point of the current radical and the last point of the previous radical
Stroke writing latency	Duration between the offset of the last stroke to onset of the current stroke
Stroke writing duration	Difference between the timestamps of the last and first stroke writing samples
Stroke writing length	Sum of the lengths of the strokes
Stroke average pen pressure	Mean pressure of the pen samples in the stroke
Stroke distance	Linear distance between the current stroke’s first sample and the previous stroke’s last sample

Taken together, OpenHandWrite_Toolbox delivers a complete pipeline for handwriting research, spanning experiment design, data collection, character segmentation, and feature extraction. This integrated package enables researchers to conduct fine-grained analyses of handwriting behavior and investigate its cognitive underpinnings.

### Development of a multi-level database of Chinese character handwriting

We used OpenHandWrite_Toolbox to collect handwriting data through a handwriting-to-dictation task. Participants used an inking digitizer pen to write target characters as quickly and accurately as possible, following spoken prompts. After writing, the target character appeared on screen, prompting participants to self-report whether their writing was correct, incorrect due to character amnesia (i.e., a character amnesia response), or incorrect because they did not know which character they were supposed to handwrite (a “don’t know” response). OpenHandWrite_Toolbox was used to present stimuli and record handwriting data.

#### Participants

Forty-two native adult Mandarin speakers were recruited in this study (32 females; mean age = 20.50 years; range = 19–22 years). All were right-handed according to the Edinburgh Handedness Questionnaire (Oldfield, 1971), with normal hearing and normal or corrected-to-normal vision. None reported neurological or psychiatric disorders. All participants provided written consent before participating. An ethics committee of The Chinese University of Hong Kong approved the protocol.

#### Materials

Characters were selected from the Chinese film subtitle corpus (SUBTLEX-CH; Cai & Brysbaert, 2010). As one of the purposes was to examine character amnesia in Chinese handwriting, we applied several filters to select a total of 1200 characters. Selection criteria included log frequency between 1.5 and 5.0 (corresponding to 32–100,000 corpus occurrences) and more than four strokes to avoid ceiling effects. These filters resulted in a candidate set of 2095 characters. For each character, we extracted the most common bi-character word from the SUBTLEX-CH (e.g., 水稻*, shui*_*3*_* dao*_*4*_, for target character 稻, *dao*_*4*_). These words were then rated for familiarity by 15 participants on a scale from 1 to 7 (the highest value represents the most familiar). We only included items where the context word was rated 4 or above for familiarity. We randomly selected 1200 characters that conformed to all the above filtering criteria. These characters were then compiled into dictation phrases (e.g., 水稻的稻, the target character 稻, meaning “rice”, in the word 水稻, meaning “wetland rice”). The audio recordings of the dictation phrases were then generated using Google Text-to-Speech (https://cloud.google.com/text-to-speech).

#### Lexical variables

All lexical variables for the selected characters are described in Wang et al. (2020). Each character was characterized by 14 phonological, semantic, and orthographic factors. We used the following four phonological variables. *Phonograms* refer to whether a character is a phonogram according to the *Dictionary of Modern Chinese Phonograms* (现代汉字形声字字汇; Ni, 1982; a character was coded as 0.5 if it is a phonogram and – 0.5 if it is not). *Phonetic* *radical order* refers to whether a character has a phonetic radical that is written first (coded as 0.5 if it does and – 0.5 if it does not; non-phonograms were categorized as not having the phonetic radical first). *Character regularity* refers to the extent to which the character contained a phonetic radical that indicated the pronunciation of the character. This was adopted from Wang et al. (2020). In their study, 53 native Mandarin speakers each rated 400 characters randomly selected from a 1600-character pool. On each trial, a written character was presented, and participants could listen to its pronunciation if needed. They then rated the extent to which the character contained a phonetic radical indicating its pronunciation (0 = not at all, 7 = containing an identically sounding radical). *Homophone density* refers to the number of characters that have the same pronunciation as a target character. There were three semantic variables. *Number of meanings* refers to the number of meanings a character has according to *Xinhua Dictionary* (11th edition, Linguistics Institute of the Chinese Academy of Social Sciences, 2011). *Imageability* and *concreteness* refer to the perceived imageability and concreteness of a character as determined by subjective ratings. Finally, we also considered the following five orthographic variables. *Character* *frequency* refers to the log of a character’s count in the Chinese subtitle corpus SUBTLEX-CH (Cai & Brysbaert, 2010). *Age of acquisition* refers to the age a child learns a character according to the objective age of acquisition of Chinese characters in Cai et al. (2021) and Shu et al. (2003). *Number of strokes* refers to the number of strokes in a character according to the *Dictionary of Common Chinese Characters* in Print (印刷通用汉字字形表; Chinese Ministry of Culture & State Language Affairs Commission, 1986) and the *Modern Dictionary of Common Characters* in Chinese (现代汉语通用字表; Chinese Ministry of Culture & State Language Affairs Commission, 1988). *Number of radicals* refers to the number of radicals in a character according to the *Dictionary of Chinese Character Properties* (汉字属性字典; Fu, 1989), which defines radicals as orthographic components, independent of whether they function as phonetic or semantic radicals. *Character* *composition* refers to the organization of radicals within a character according to the *Dictionary of Chinese Character Properties*, it was sum-coded into two variables: whether a character has a left-right composition (coded as 0.5) or not (coded as – 0.5), and whether a character has a top-down composition (coded as 0.5) or not (coded as – 0.5). *Context* *word familiarity* refers to the familiarity of the context words.

#### Apparatus and procedure

**Fig. 6.**
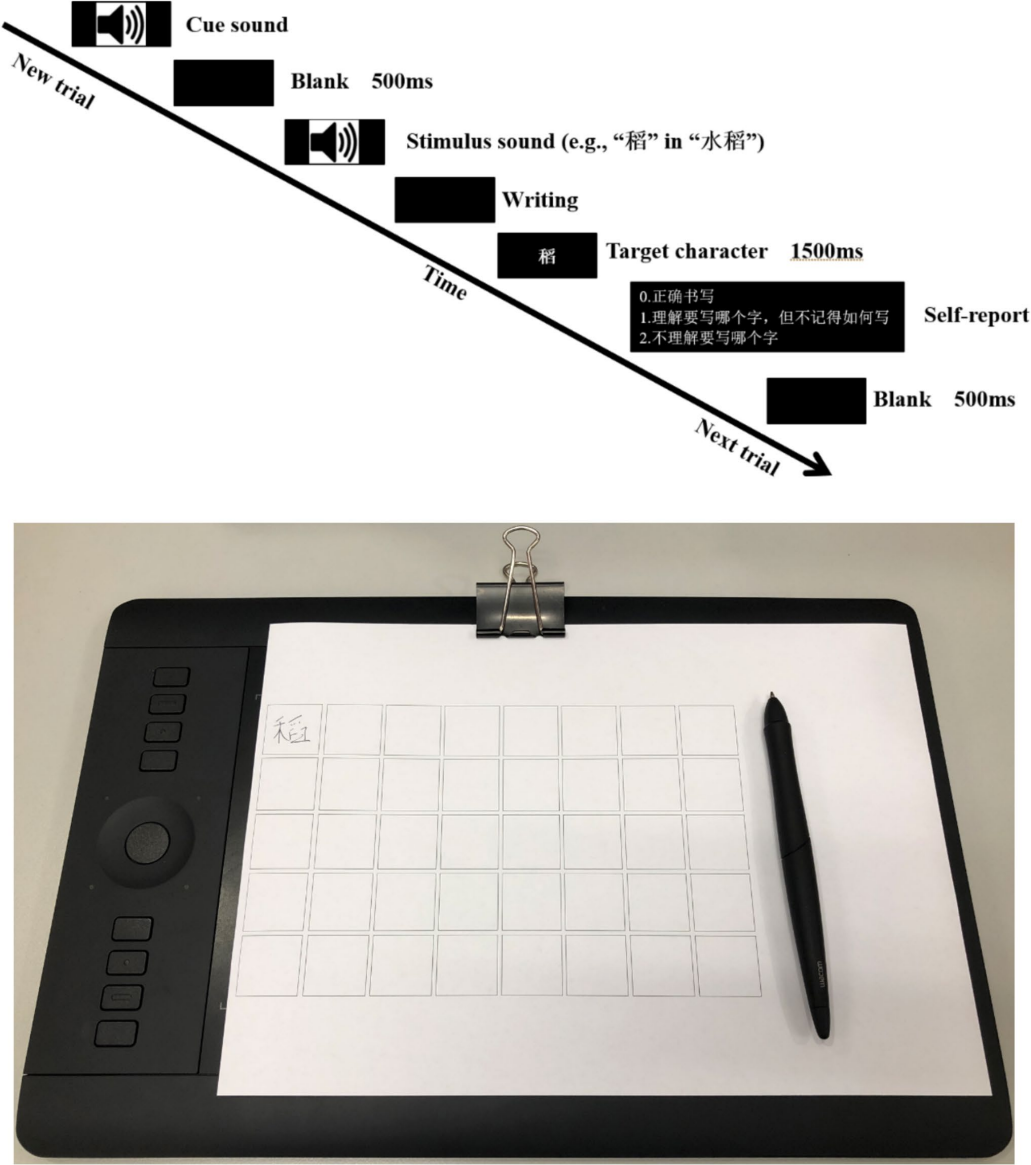
***Upper panel***: An example trial of the handwriting-to-dictation task. On a trial, participants heard a cue sound and, 500 ms later, a dictation prompt specifying a target character. They handwrote the target character as quickly and accurately as possible. After participants finished handwriting, they were shown the target character on screen and self-reported whether they had a correct response, a character amnesia response, or a don’t know response. There was a 500-ms inter-trial interval with a blank screen. ***Lower panel***: An example of the sheet of 8 × 5 grid paper on top of the tablet

We adopted a handwriting-to-dictation task (Fig. 6, upper panel) where participants first heard a cue sound, signaling the start of the experimental trial, followed by a 500-ms blank interval. At this stage, participants were instructed to hold the pen above the correct grid square. They then heard a dictation prompt specifying a character to be handwritten (e.g., 水稻的稻, *shui*_*3*_* dao*_*4*_* de dao*_*4*_, the target character “稻” in the word “水稻”). Participants used an inking digitizer pen (Wacom KP-130-00DB) to write the target character in a grid on an 8 × 5 grid sheet affixed to a Wacom Intuos tablet (Wacom PTH-651; sampling rate set at 200 Hz), as quickly and accurately as possible after the offset of the audio.

In case they did not know how to write the character, they were instructed to draw a circle in the grid. They then pressed the spacebar on the keyboard to indicate the completion of handwriting, which triggered the presentation of the target character for 1500 ms. Participants used the pen tip to press a key to self-report whether they had written the correct character (a correct handwriting response, number key 0), whether they knew which character was supposed to write but had forgotten how to write it (a character amnesia response, number key 1), or whether they did not know which character was supposed to write (a don’t know response, number key 2), followed by a 500-ms blank interval. Each participant took part in three experimental sessions; the interval between sessions was chosen by participants, ranging from 2 h to 4 days. In each session, participants handwrote 400 characters, with 40 characters on each 8 × 5 grid paper from left to right, top to bottom (Fig. 6, lower panel). The experiment automatically paused after dictating 40 characters, allowing the experimenter to replace the paper sheet.

#### Data coding

Three helpers manually checked the penscripts (i.e., the collected handwritten images) against participants’ self-reports on the online survey platform Qualtrics (https://www.qualtrics.com/). The helpers were first shown a target character in its typed format, followed by the 42 corresponding penscripts of the character (from 42 participants). Each of them checked 16,800 penscripts (of 400 target characters). They were instructed to mark a penscript as “correct handwriting” if they recognized it as the target character (i.e., 44065 responses; 87.4% of the trials) and “incorrect handwriting” otherwise (i.e., 4950 responses; 9.8% of the trials). Additionally, they were instructed to indicate if a penscript had been revised (e.g., with a false start, with a crossed character or radical), with a total of 1385 penscripts (2.7%) eventually categorized as “revised”. Incorrect and revised penscripts were subsequently excluded from the analyses of handwriting latency, duration, and pen pressure. This manual checking was also conducted to validate the self-reports on character amnesia rather than to independently define amnesia trials. We expected that trials with a “correct handwriting” self-report always had a correctly handwritten character (from the raters) and trials with a “character amnesia” or “don’t-know” self-report always had an incorrectly handwritten character or a missing handwriting. Overall, 97.62% of trials showed consistency between participants’ self-reports and the manually checked penscripts, indicating high reliability of the self-report data. In addition, we evaluated the inter-rater reliability of the manual coding. Another 12 helpers were recruited to check for inter-rater reliability. They were randomly assigned to three groups of four raters, each group coded all the penscripts, such that each penscript was coded by three different helpers. We conducted an intraclass correlation analysis to assess coding inter-rater reliability. The results revealed an intraclass correlation coefficient (ICC) of 0.829, with a 95% confidence interval for ICC population values ranging from 0.827 to 0.831, indicating strong agreement among raters.

#### Data exclusion

At the character level, we removed any writing latencies longer than 10 s and writing durations shorter than 1 s or longer than 10 s (Wang et al., 2020), leading to the exclusion of 0.39% of the writing latency data and 3.33% of the writing duration data. At the radical level, we removed latencies and durations longer than 2 s, leading to the exclusion of 0.28% of the writing latency data and 0.03% of the writing duration data. At the stroke level, we removed latencies and durations longer than 2 s, leading to the exclusion of 0.07% of the writing latency data and 0.02% of the writing duration data.

## Results

**Fig. 7:**
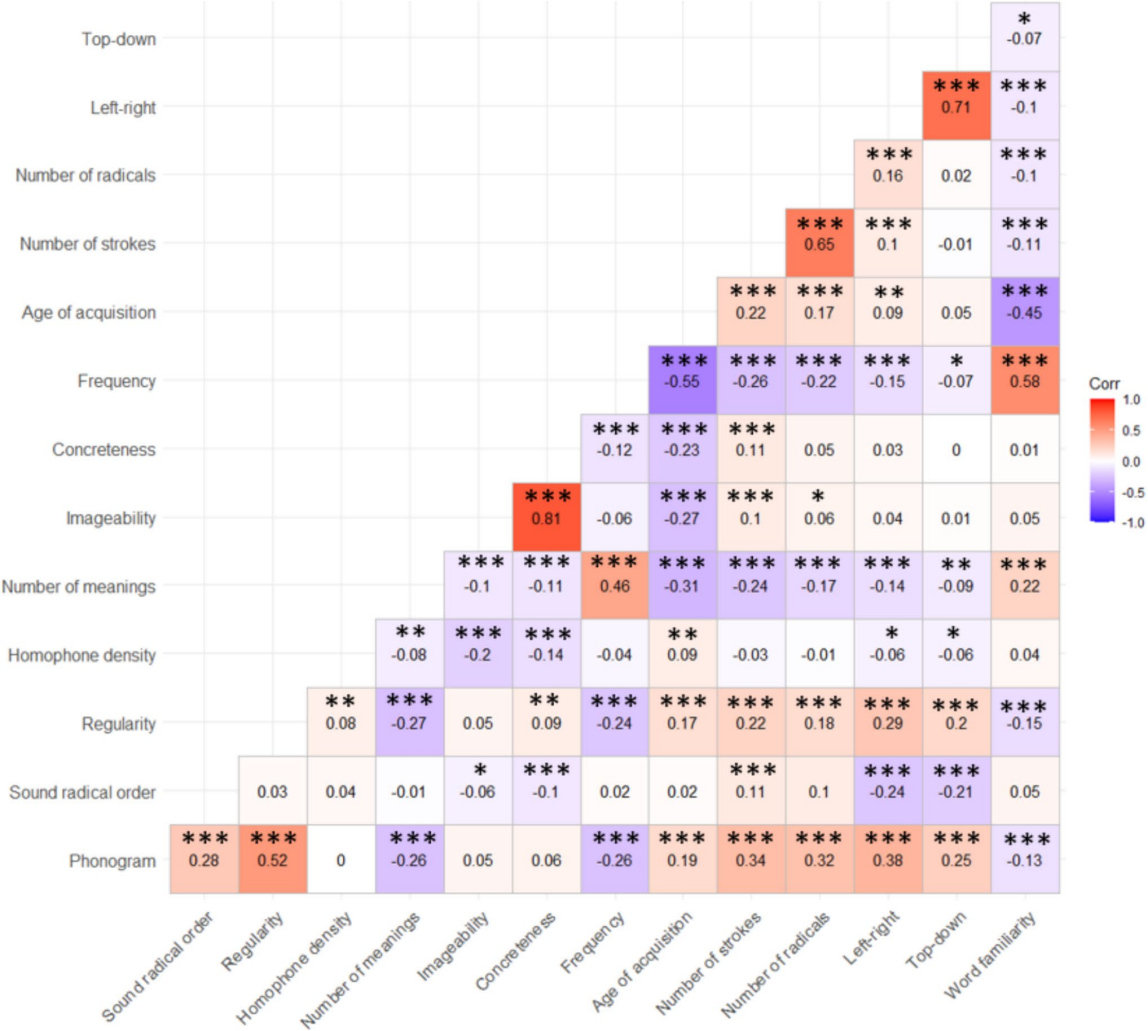
Correlations of all variables (phi-correlation for binary variables). All the values listed within each matrix indicate correlation coefficients between variables (*** Correlation is significant at the.001 level; ** Correlation is significant at the.01 level; * Correlation is significant at the.05 level)

Following previous large-scale studies on Chinese character recognition (Tsang et al., 2018; Sze et al., 2015), naming (Chang et al., 2016; Liu et al., 2007), and handwriting (Wang et al., 2020), we conducted item-level analyses by collapsing handwriting responses across participants and computing the mean performance for each unique character across participants. Radical-level and stroke-level handwriting measures were aggregated within each character. Specifically, for characters containing multiple radicals, radical-level handwriting measures were computed by averaging the latency and duration measures across all radicals within the target character. A similar aggregation procedure was applied at the stroke level. The rationale for adopting this aggregation strategy was to maintain the character as the unit of analysis across all three orthographic levels. This allows us to address the research question of how each character’s lexical variables influence handwriting across different levels. Table 2 presents descriptive results for the lexical variables and handwriting measures. Figure 7 represents the correlations (Person’s *r*) among the variables for the 1200 Chinese characters. All stimuli and trial-level data are available on the Open Science Framework (https://osf.io/rn2ck/?view_only=4e2fc80957314e53a2afd47a1db8f217).

**Table 2:** Descriptive results for lexical variables, character-, radical-, and stroke-level handwriting data

Variable	Mean	SD	Range
Phonogram	0.75	0.43	– 0.5 to 0.5
Phonetic radical order	0.18	0.39	– 0.5 to 0.5
Regularity	– 0.01	0.85	– 1.30–1.72
Homophone density	0.90	0.35	0.00–1.72
Number of meanings	3.20	2.10	1.00–16.00
Imageability	– 0.01	0.60	– 1.91 to 1.37
Concreteness	– 0.02	0.69	– 1.69 to 1.56
Frequency	3.44	0.68	1.58–5.00
Age of acquisition	8.50	1.63	6.50–15.00
Number of strokes	9.72	2.96	5.00–21.00
Number of radicals	2.95	1.04	1.00–7.00
Left-right	0.58	0.49	– 0.5 to 0.5
Top-down	0.73	0.45	– 0.5 to 0.5
Word familiarity	0.33	0.33	– 1.19 to 1.21
Character latency	1031.80	485.15	297.95–3861.67
Character duration	2222.05	656.29	1134.86–5011.30
Character pressure	12,549.66	856.97	8779.62–15,818.14
Character length	40.61	8.48	19.61–68.40
Character amnesia rate	0.05	0.08	0.00–0.59
Radical latency	151.30	45.01	61.38–547.05
Radical duration	707.17	310.87	104.05–2331.60
Radical pressure	12,856.35	1211.96	6405.25–18,084.43
Radical length	15.44	5.89	4.03–41.85
Radical distance	3.83	1.28	0.78–8.83
Stroke latency	113.11	28.68	78.86–398.69
Stroke duration	147.93	28.07	88.01–314.02
Stroke pressure	12,096.85	1009.73	8323.09–16,189.78
Stroke length	5.14	1.17	2.29–10.88
Stroke distance	2.51	0.50	1.20–5.29

Homophone density refers to the log of the number of homophonous characters in SUBTLEX-CH. Regularity, imageability, concreteness, and context word familiarity were based on ratings that were individually normalized. Frequency refers to the log of character counts in SUBTLEX-CH. Handwriting data are reported in milliseconds

We next provide some analyses of the database to investigate how handwriting performance is influenced by the lexical variables. As there were correlations among the variables (shown in Fig. 6), we assessed collinearity issues using stepwise variance inflation factor (VIF) selection (using the R package fmsb). All variables remained below the VIF threshold of 5 (Wang et al., 2020), indicating no serious collinearity among these lexical variables. We conducted multiple regression models to examine how different handwriting measures (character amnesia, handwriting latency, and handwriting duration) are influenced by the lexical variables. The analyses included the following lexical variables (all *z*-transformed and entered): phonogram, phonetic radical order, regularity, homophone density, number of meanings, imageability, concreteness, frequency, age of acquisition, number of strokes, number of radicals, left-right composition, top-down composition, and word familiarity. To account for multiple comparisons across lexical variables, we applied a false discovery rate (FDR; Benjamini & Hochberg, 1995) correction with a threshold of *p* <.05 in all regression analyses.

Split-half reliability was assessed to evaluate the stability of item-level estimates in the database. Participants were randomly divided into two equal groups (21 per group). For each half, item-level measures were computed separately for character amnesia rate, character-level handwriting latency, and character-level handwriting duration. Pearson correlations were then calculated across characters between the two halves. The results showed strong and significant correlations (corrected using the Spearman–Brown prophecy formula) for character-level handwriting latency (*r* = 0.95, *p* <.001, *N* = 1200), character-level handwriting duration (*r* = 0.80, *p* <.001, *N* = 1200), and character amnesia rate (*r* = 0.88, *p* <.001, *N* = 1200), indicating good reliability of the item-level measures. Additionally, we conducted correlational analyses of the character-level results between our database and those of Wang et al. (2020). The results showed significant correlations for character writing latencies (*r* = 0.74, *p* < 0.001, *N* = 1200) and character writing durations (*r* = 0.94, *p* < 0.001, *N* = 1200) between two databases.

### Character amnesia rate

The results of the regression model (*R*^2^ =.420) are presented in Table 3. The results show that phonological factors influence a character’s tendency for amnesia: Characters are more likely to result in amnesia when the first radical is a phonetic radical or when they have lower regularity. Semantic predictors do not impact character amnesia rate. Orthographic factors modulate amnesia rate: Character amnesia rates are higher for characters that are less frequent, learned later, contain more strokes, do not have a top-down composition, or appear in less familiar contextual words.

**Table 3:** Results of character-level regressions on amnesia rate, writing latency, and writing duration

	Amnesia rate			Character latency			Character duration		
Linear term	*β*	*t*	*p*	*β*	*t*	*p*	*β*	*t*	*p*
(Intercept)	0.057	16.32	<.001***	1043.903	58.30	<.001***	1148.852	19.31	<.001***
Phonogram	– 0.009	– 1.40	0.205	1.416	0.04	0.965	– 44.536	– 2.40	0.047*
Phonetic radical order	0.017	2.85	0.011*	71.385	2.39	0.034*	27.058	1.57	0.212
Regularity	– 0.007	– 2.72	0.013*	– 3.452	– 0.28	0.837	– 3.077	– 0.44	0.711
Homophone density	– 0.003	– 1.42	0.205	25.764	2.46	0.033*	– 3.085	– 0.51	0.709
Number of meanings	– 0.001	– 0.41	0.683	11.878	1.01	0.441	– 5.695	– 0.84	0.510
Imageability	– 0.006	– 1.84	0.116	– 12.703	– 0.72	0.597	13.321	1.33	0.288
Concreteness	0.004	1.09	0.323	– 48.257	– 2.75	0.017*	– 9.212	– 0.92	0.503
Frequency	– 0.022	– 7.59	<.001***	– 158.005	– 10.55	<.001***	– 54.729	– 6.36	<.001***
Age of acquisition	0.028	10.70	<.001***	121.499	9.03	<.001***	35.363	4.56	<.001***
Number of strokes	0.015	5.61	<.001***	51.338	3.69	<.001***	428.329	34.83	<.001***
Number of radicals	0.002	0.63	0.573	– 6.365	– 0.47	0.745	– 0.061	– 0.01	0.994
Left-right	– 0.011	– 1.78	0.117	– 73.977	– 2.32	0.036*	– 46.019	– 2.42	0.047*
Top-down	– 0.018	– 2.84	0.011*	– 66.401	– 2.02	0.067	30.505	1.55	0.212
Word familiarity	– 0.013	– 5.34	<.001***	– 105.341	– 8.26	<.001***	– 15.532	– 2.13	0.079

Key: *β* = coefficient, *t* = *t*-value, *p* = *p* value. Significant *p* values are indicated in bold

The result of character duration has been accounted for by character length, stroke distance, and stroke writing latency

### Character writing latency

The results of the regression model (*R*^*2*^ =.482) are presented in Table 3. Phonology modulates character writing preparation time: writing latencies are shorter when the first radical is not a phonetic radical, or when characters have lower homophone density. Semantic predictors impact writing preparation time; characters with higher concreteness predict shorter latencies. Orthographically, characters with higher frequency, when learned at an earlier age, with fewer strokes, have a left-right composition, or are embedded in the more familiar words, are associated with shorter writing latencies.

### Character writing duration

The results of the regression model (*R*^*2*^ =.907) are presented in Table 3. Phonology modulates character writing duration: writing duration is shorter when the character is a phonogram. Orthography modulates character writing durations: they are shorter for characters with higher frequency, earlier age of acquisition, fewer strokes, or with a left-right composition. Semantic predictors do not significantly affect writing durations.

### Radical writing latency

The results of the regression model (*R*^*2*^ =.291) are presented in Table 4. Phonology influences radical writing preparation time: writing latency is shorter when a character’s phonetic radical does not appear first. Orthography also modulates radical writing preparation time: Shorter radical writing latencies are associated with characters that have higher frequency, are learned earlier, contain fewer strokes, have more radicals, or have a left-right composition. Semantic predictors do not modulate writing preparation time.

**Table 4:** Results of radical-level regressions on writing latency and writing duration

	Radical latency			Radical duration		
Linear term	*β*	*t*	*p*	*β*	*t*	*p*
(Intercept)	119.301	27.52	<.001***	– 153.471	– 6.86	<.001***
Phonogram	– 5.338	– 1.45	0.205	29.953	2.95	0.009**
Phonetic radical order	13.961	4.17	<.001***	– 35.183	– 3.85	0.001**
Regularity	0.303	0.23	0.822	– 2.774	– 0.75	0.638
Homophone density	– 1.532	– 1.30	0.245	– 3.182	– 0.98	0.576
Number of meanings	– 2.050	– 1.56	0.205	4.032	1.10	0.576
Imageability	2.890	1.49	0.205	4.107	0.76	0.638
Concreteness	1.769	0.91	0.424	– 5.592	– 1.04	0.576
Frequency	– 7.408	– 4.48	<.001***	– 3.050	– 0.66	0.647
Age of acquisition	7.500	5.08	<.001***	2.377	0.58	0.657
Number of strokes	10.628	6.94	<.001***	82.252	17.75	<.001***
Number of radicals	– 5.866	– 3.87	<.001***	– 22.399	– 4.18	<.001***
Left-right	– 11.249	– 2.97	0.007**	– 39.474	– 3.59	0.001**
Top-down	– 2.484	– 0.60	0.591	4.663	0.41	0.703
Word familiarity	– 2.220	– 1.55	0.205	– 1.521	– 0.38	0.703

Key: *β* = coefficient, *t* = *t*-value, *p* = *p* value. Significant *p* values are indicated in bold

The result of radical latency has been accounted for by radical distance; radical duration has been accounted for by radical length and stroke writing latency

### Radical writing duration

The results of the regression model (*R*^*2*^ =.866) are presented in Table 4. Phonology influences radical writing durations, with shorter durations for non-phonogram characters than for phonograms and for characters with a phonetic radical to be written first than others. Orthographically, there are shorter radical writing durations for characters that have fewer strokes, have more radicals, or have a left-right composition. Semantic predictors do not modulate writing durations.

### Stroke writing latency

The results of the regression model (*R*^*2*^ =.173) are presented in Table 5. Orthography influences stroke writing latencies: preparation time is shorter for characters that have higher frequency, are learned earlier, have a left-right composition, with a top-down composition. Neither phonological nor semantic factors significantly affect stroke writing latency.

**Table 5:** Results of stroke-level regressions on writing latency and writing duration

	Stroke latency			Stroke duration		
Linear term	*β*	*t*	*p*	*β*	*t*	*p*
(Intercept)	70.309	16.12	<.001***	52.016	17.39	<.001***
Phonogram	0.236	0.10	0.923	– 2.885	– 2.42	0.055
Phonetic radical order	– 1.063	– 0.47	0.782	0.556	0.50	0.782
Regularity	– 0.752	– 0.82	0.642	0.644	1.44	0.264
Homophone density	0.738	0.94	0.612	– 0.449	– 1.17	0.342
Number of meanings	0.352	0.40	0.782	– 0.509	– 1.17	0.342
Imageability	0.462	0.35	0.782	1.120	1.74	0.164
Concreteness	1.361	1.03	0.612	– 1.399	– 2.17	0.084
Frequency	– 3.471	– 3.08	0.007**	0.148	0.27	0.889
Age of acquisition	3.299	3.26	0.005**	0.109	0.22	0.889
Number of strokes	0.630	0.60	0.772	5.702	10.27	<.001***
Number of radicals	2.002	1.93	0.152	0.044	0.09	0.931
Left-right	– 22.340	– 9.31	<.001***	– 2.845	– 2.42	0.055
Top-down	– 19.427	– 7.83	<.001***	3.273	2.59	0.055
Word familiarity	0.907	0.95	0.612	– 0.876	– 1.87	0.144

Key: *β* = coefficient, *t* = *t*-value, *p* = *p* value. Significant *p* values are indicated in bold

The result of stroke latency has been accounted for stroke distance; radical duration has been accounted for stroke length and stroke distance

### Stroke writing duration

The results of the regression model (*R*^*2*^ =.795) are presented in Table 5. Phonology and semantics do not influence stroke durations. Orthographically, characters with fewer strokes are associated with shorter stroke handwriting durations.

### Hierarchical linguistic effects across different levels of writing latency

**Fig. 8:**
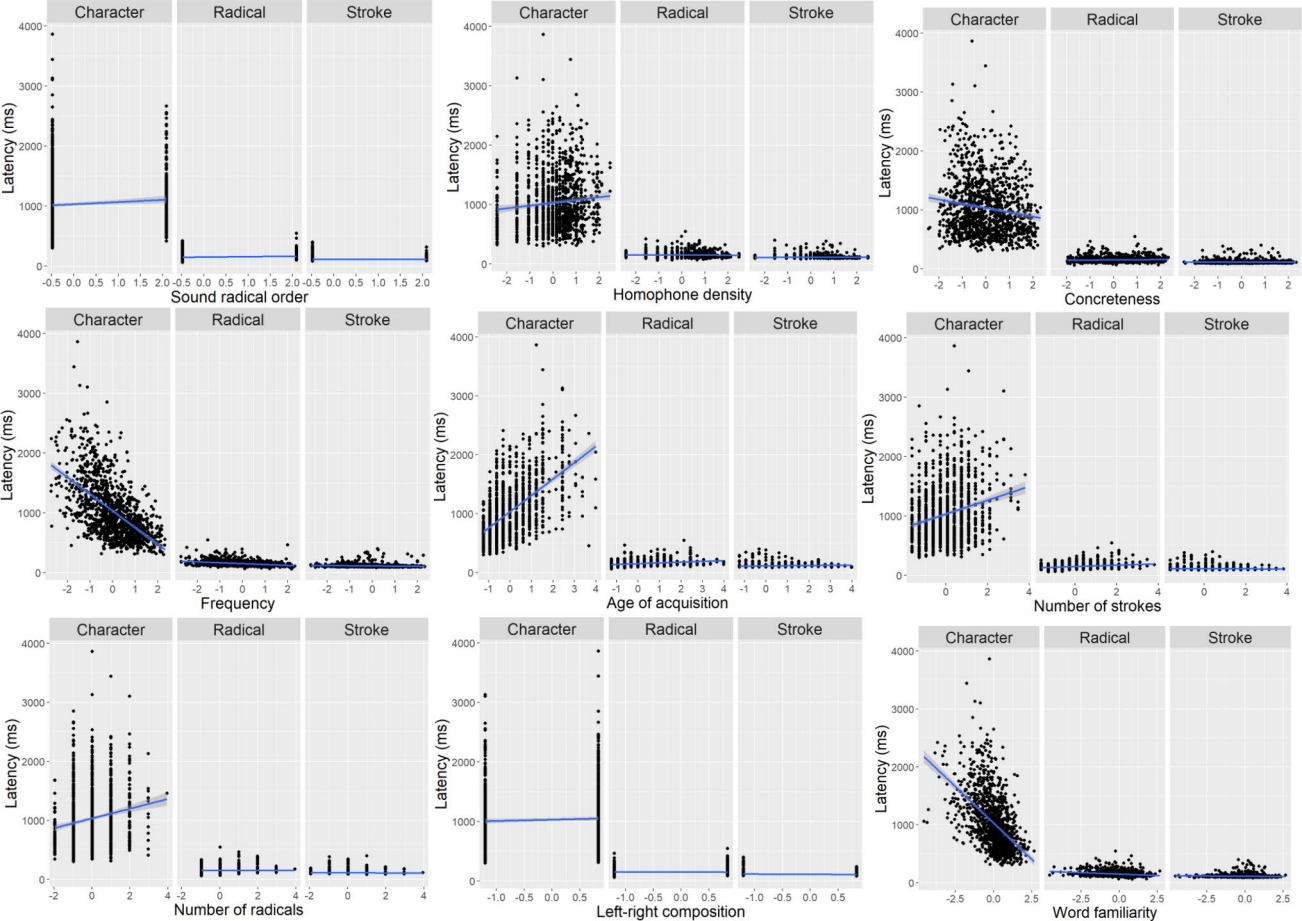
Significant interaction effects of writing latencies as a function of lexical variables, as described in Table 6, including phonetic radical order, homophone density, concreteness, frequency, age of acquisition, number of strokes, number of radicals, left-right composition, and word familiarity

Latencies are the longest at the character level, followed by those at the radical level, and are the shortest at the stroke level. The effects of the following lexical variables on the increase of latencies are larger at the character level compared to either the radical level or the stroke level: phonetic radical to be written first, more homophone density, lower concreteness, lower frequency, later age of acquisition, more strokes, or less word familiarity. Compared with stroke latencies, radical latencies have a greater increase for characters whose first radical is not a phonetic radical, have more strokes, fewer radicals, or a left-right composition (Table 6 and Fig. 8).

**Table 6:** Results of character-, radical-, and stroke-level regressions on writing latency

	Level: Character vs. Radical			Level: Character vs. Stroke			Level: Radical vs. Stroke		
Linear term	*β*	*t*	*p*	*Β*	*t*	*p*	*β*	*t*	*p*
(Intercept)	603.58	65.04	<.001***	576.57	64.20	<.001***	133.25	106.49	<.001***
Level	892.72	48.10	<.001***	934.67	52.04	<.001***	47.94	19.16	<.001***
Phonogram	– 2.75	– 0.16	0.870	0.64	0.04	0.969	– 3.53	– 1.56	0.210
Phonetic radical order	45.69	2.99	0.008**	34.52	2.30	0.033*	8.83	4.28	0.002**
Regularity	– 1.25	– 0.20	0.870	– 2.26	– 0.37	0.767	– 0.06	– 0.08	0.940
Homophone density	12.16	2.25	0.039*	13.31	2.53	0.019*	– 0.27	– 0.37	0.767
Number of meanings	4.87	0.80	0.539	5.94	1.00	0.442	– 1.01	– 1.23	0.308
Imageability	– 5.01	– 0.56	0.674	– 6.15	– 0.70	0.617	1.49	1.23	0.308
Concreteness	– 23.45	– 2.61	0.021*	– 23.50	– 2.67	0.016*	1.11	0.91	0.461
Frequency	– 82.43	– 10.73	<.001***	– 80.67	– 10.75	<.001***	– 5.48	– 5.29	<.001***
Age of acquisition	64.51	9.31	<.001***	62.54	9.27	<.001***	5.49	5.88	<.001***
Number of strokes	32.08	4.54	<.001***	25.23	3.62	<.001***	6.26	6.57	<.001***
Number of radicals	– 8.54	– 1.25	0.297	– 3.16	– 0.47	0.749	– 5.44	– 5.89	<.001***
Left-right	– 42.98	– 2.54	0.023*	– 48.71	– 3.05	0.006**	– 17.71	– 7.75	<.001***
Top-down	– 40.33	– 2.29	0.039*	– 44.08	– 2.68	0.016*	– 18.01	– 7.58	<.001***
Word familiarity	– 53.95	– 8.19	<.001***	– 52.13	– 8.15	<.001***	– 0.62	– 0.70	0.566
Level×Phonogram	3.54	0.25	0.804	0.68	0.05	0.962	– 2.88	– 1.49	0.189
Level×Phonetic radical order	20.12	1.68	0.163	28.59	2.46	0.033*	8.75	5.42	<.001***
Level×Regularity	– 4.38	– 0.35	0.784	– 2.38	– 0.20	0.911	2.01	1.19	0.271
Level×Homophone density	27.13	2.51	0.028*	24.89	2.37	0.036*	– 2.28	– 1.56	0.185
Level×Number of meanings	13.78	1.13	0.360	11.87	1.00	0.443	– 2.03	– 1.24	0.271
Level×Imageability	– 15.18	– 0.84	0.508	– 13.11	– 0.75	0.581	2.18	0.90	0.398
Level×Concreteness	– 48.85	– 2.72	0.023*	– 49.50	– 2.82	0.014*	– 0.27	– 0.11	0.910
Level×Frequency	– 149.66	– 9.74	<.001***	– 154.60	– 10.3	<.001***	– 4.25	– 2.05	0.104
Level×Age of acquisition	114.28	8.25	<.001***	117.86	8.73	<.001***	3.76	2.01	0.104
Level×Number of strokes	37.41	2.65	0.023*	52.20	3.74	<.001***	14.24	7.47	<.001***
Level×Number of radicals	4.76	0.35	0.784	– 6.42	– 0.47	0.743	– 10.97	– 5.95	<.001***
Level×Left-right	– 30.43	– 1.83	0.135	– 24.93	– 1.58	0.199	5.62	2.51	0.043*
Level×Top-down	23.12	1.48	0.216	19.74	1.36	0.273	– 3.33	– 1.58	0.185
Level×Word familiarity	– 103.29	– 7.84	<.001***	– 106.38	– 8.32	<.001***	– 3.37	– 1.90	0.116

Key: *β* = coefficient, *t* = *t*-value, *p* = *p* value. Significant *p* values are indicated in bold

### Hierarchical linguistic effects across different levels of writing duration

**Fig. 9:**
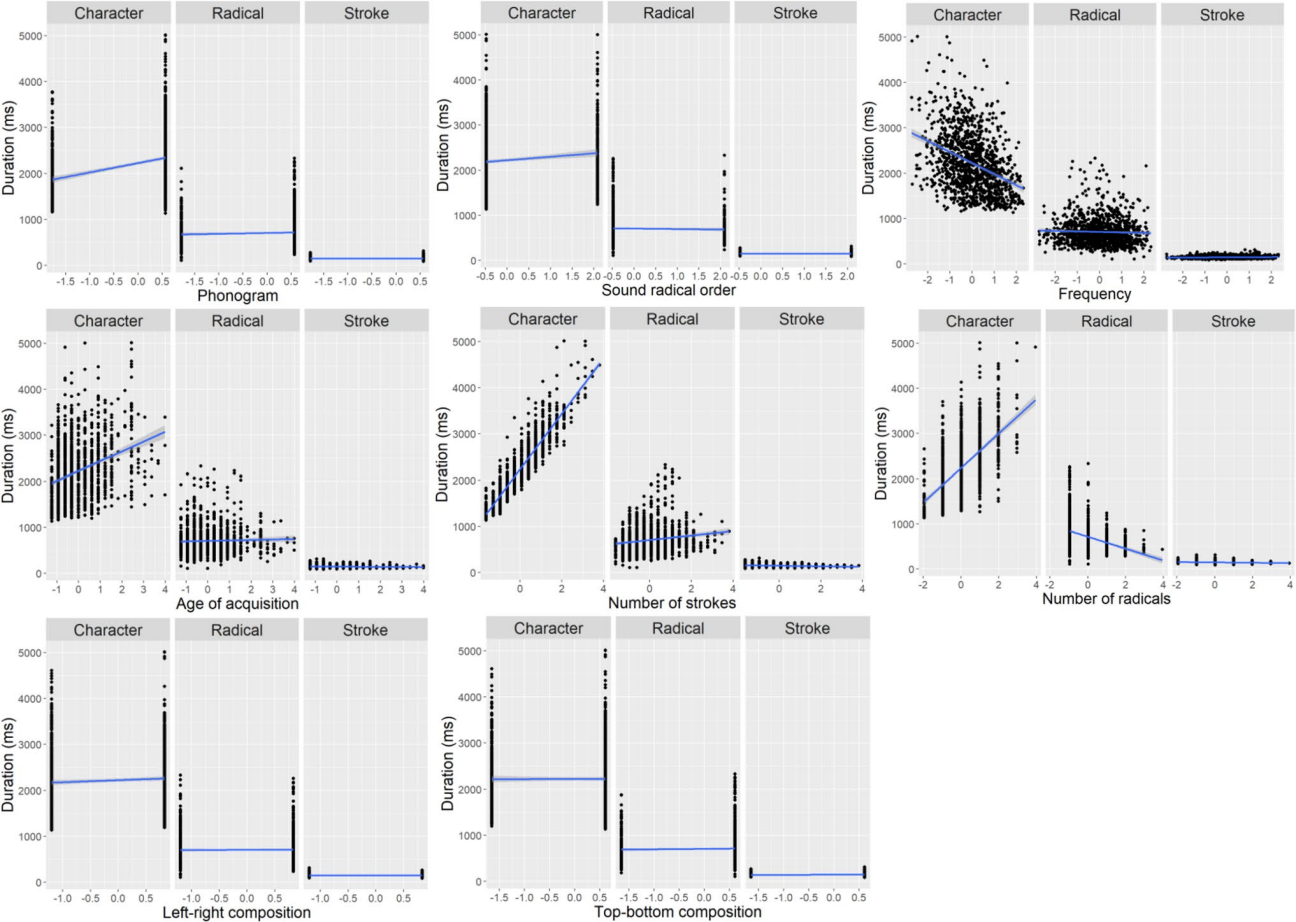
Significant interaction effects of writing durations as a function of lexical variables, as described in Table 7, including phonogram, phonetic radical order, frequency, age of acquisition, number of strokes, number of radicals, left-right composition, and top-down composition

Durations are longest at the character level, followed by those at the stroke level, and are shortest at the stroke level (after controlling for the length of writing). Compared with the character writing durations, the decrease in radical writing durations is larger for characters with more radicals. Compared with radical and stroke writing durations, character durations have a greater increase for characters that have a lower frequency, have a later age of acquisition, and contain more strokes. Compared with the radical writing durations, the increase of character writing durations is larger for characters that are not phonograms, and with phonetic radicals to be written first. Compared with the stroke durations, the increase in character durations is larger for characters that do not have a left-right composition. Compared with stroke writing durations, radical durations have a larger increase for characters that are phonograms, with a phonetic radical to be written later, with more strokes, contain fewer radicals, do not have a left-right composition, or have a top-down composition (Table 7 and Fig. 9).

**Table 7:** Results of character-, radical-, and stroke-level regressions on writing duration

	Level: Character vs. Radical			Level: Character vs. Stroke			Level: Radical vs. Stroke		
Linear term	*β*	*t*	*p*	*Β*	*t*	*p*	*Β*	*t*	*p*
(Intercept)	646.48	26.25	<.001***	671.01	29.37	<.001***	– 4.69	– 0.59	0.599
Level	1366.95	27.75	<.001***	1287.39	28.18	<.001***	– 64.62	– 4.08	0.006**
Phonogram	– 6.76	– 0.59	0.645	– 23.44	– 2.39	0.048*	14.01	2.60	0.025*
Phonetic radical order	– 7.57	– 0.73	0.635	12.02	1.33	0.324	– 18.37	– 3.74	<.001***
Regularity	– 4.78	– 1.13	0.401	– 2.14	– 0.58	0.604	– 1.84	– 0.92	0.500
Homophone density	– 1.37	– 0.37	0.765	– 0.84	– 0.27	0.790	– 1.05	– 0.60	0.588
Number of meanings	0.22	0.05	0.958	– 2.61	– 0.73	0.604	2.49	1.28	0.313
Imageability	8.87	1.45	0.263	7.81	1.47	0.282	2.29	0.79	0.545
Concreteness	– 4.13	– 0.68	0.635	– 3.73	– 0.71	0.604	– 1.77	– 0.62	0.588
Frequency	– 36.00	– 6.91	<.001***	– 31.67	– 7.01	<.001***	– 4.39	– 1.78	0.150
Age of acquisition	25.40	5.40	<.001***	21.72	5.34	<.001***	3.59	1.62	0.186
Number of strokes	251.87	40.00	<.001***	214.91	39.07	<.001***	45.92	18.77	<.001***
Number of radicals	– 9.13	– 1.65	0.229	2.66	0.65	0.604	– 10.54	– 4.04	<.001***
Left-right	– 92.88	– 8.05	<.001***	– 52.79	– 5.47	<.001***	– 42.18	– 7.73	<.001***
Top– down	– 25.60	– 2.12	0.095	– 6.10	– 0.60	0.604	– 14.50	– 2.52	0.028*
Word familiarity	– 6.45	– 1.44	0.263	– 7.10	– 1.85	0.152	– 0.32	– 0.15	0.881
Length	34.87	43.66	<.001***	23.41	10.29	<.001***	35.39	28.68	<.001***
Level×Phonogram	– 31.78	– 3.28	0.004**	– 18.05	– 2.12	0.095	14.15	3.10	0.005**
Level×Phonetic radical order	23.79	2.93	0.010*	8.37	1.19	0.408	– 15.34	– 3.99	<.001***
Level×Regularity	– 0.59	– 0.07	0.955	– 5.90	– 0.80	0.593	– 5.29	– 1.32	0.260
Level×Homophone density	0.41	0.06	0.955	– 0.64	– 0.10	0.920	– 1.05	– 0.30	0.821
Level×Number of meanings	– 10.15	– 1.23	0.383	– 4.60	– 0.64	0.661	5.60	1.44	0.235
Level×Imageability	10.91	0.89	0.521	13.27	1.25	0.408	2.26	0.39	0.821
Level×Concreteness	– 3.86	– 0.32	0.877	– 4.74	– 0.45	0.763	– 0.85	– 0.15	0.883
Level×Frequency	– 54.27	– 5.21	<.001***	– 63.50	– 7.03	<.001***	– 8.95	– 1.82	0.139
Level×Age of acquisition	36.33	3.86	0.001**	43.56	5.36	<.001***	7.30	1.65	0.175
Level×Number of strokes	333.47	26.48	<.001***	416.27	37.84	<.001***	78.43	16.03	<.001***
Level×Number of radicals	26.23	2.38	0.041*	2.92	0.36	0.776	– 23.40	– 4.49	<.001***
Level×Left-right	– 10.41	– 0.92	0.521	– 50.02	– 5.26	<.001***	– 39.35	– 7.34	<.001***
Level×Top-down	– 7.45	– 0.70	0.618	9.82	1.09	0.432	17.29	3.39	0.002**
Level×Word familiarity	– 13.61	– 1.52	0.257	– 12.23	– 1.59	0.263	1.35	0.32	0.821
Level× Length	– 23.95	– 15.00	<.001***	– 1.03	– 0.23	0.821	22.92	9.29	<.001***

Key: *β* = coefficient, *t* = *t*-value, *p* = *p* value. Significant *p* values are indicated in bold

## Discussion

In this paper, we report the construction of a large-scale database of character handwriting at different levels (character, radical, and stroke). Multiple regression analyses revealed that orthographic predictors significantly affect handwriting at all three orthographic levels during both handwriting preparation and execution. Phonological factors influence handwriting preparation and execution at the character and radical levels but not at the stroke level. Importantly, we found that most of these lexical factors showed the strongest effects at the character level, followed by the radical level, with the smallest effects at the stroke level. We also introduced an open-source package that we developed to allow researchers with limited programming experience to conduct handwriting studies across various experimental paradigms. Though our setup was designed for writing-to-dictation, it can be easily adapted for other handwriting tasks (e.g., copying). We further provided scripts for batch-processing handwriting data, including temporal, spatial, and pen pressure information at the character, radical, and stroke levels; digitalization of handwritten images, and data visualization for handwriting details.

### The role of phonology in character and sub-character handwriting

Characters with more transparent phonology–orthography mappings are less susceptible to amnesia. These results replicate the findings by Huang et al. (2021a) and Huang et al. (2021b). However, we did not observe an effect of character regularity on handwriting latency in the present study. This finding contrasts with Wang et al. (2020), who reported a significant modulation of character-level handwriting latency by regularity. Note that the current study was very similar in design and procedure to Wang et al. (2020), except that participants in the current study handwrote all test characters, whereas those in Wang et al. (2020) handwrote only a subset. It is unlikely that the discrepancy in the effect of regularity reflects such a minor difference; instead, it is more likely that the influence of phonological regularity on handwriting latency may be less robust than previously assumed. Our findings, therefore, highlight the need for further research to clarify the circumstances under which regularity influences early stages of handwriting production.

Characters without a phonetic radical in the first position show lower amnesia rates and shorter writing durations compared with those having a phonetic radical first. Similarly, phonograms (compared with non-phonograms) are also associated with shorter handwriting execution. We also found that characters with lower homophone density exhibit shorter character writing latencies. These results suggest the involvement of phonology–orthography conversion in handwriting, so that handwriters can access a character more easily when it has a phonetic radical in its conventional position (right or bottom position), when it is a phonogram (which typically displays higher transparency between phonology and orthography compared with non-phonograms), or when it has fewer competitive homophones. The neuroimaging studies also support this hypothesis: compared with drawing symbols, handwriting Chinese characters elicited greater activation in the brain regions associated with phonological and orthographical processing (e.g., SMG; Xu et al., 2025a; Rapp & Dufor, 2011; Rapp et al., 2016; Reich et al., 2003; Lee et al., 2004). In line with earlier findings (Lau, 2020a, b), our study also shows that phonological factors modulate handwriting at the radical level. For instance, there are shorter radical latencies for characters without a phonetic radical in the first place. These findings suggest that individuals continue to utilize the sub-lexical route to facilitate handwriting at the radical level.

Furthermore, interaction effects indicate that the influence of phonology is larger at the character level than at the radical or stroke level. For instance, our study found that, compared with writing latencies at both the radical and stroke levels, character latencies show a greater increase for characters with higher homophone density. This suggests a hierarchical model of handwriting production in which phonological activation is not uniformly distributed across writing units. The selection and organization of characters are heavily influenced by phonological information, while the retrieval of the radicals and strokes themselves is less susceptible to this interference. This sheds light on learning and educational practice, suggesting that teaching methods that emphasize phonology in character-level orthographic retrieval during handwriting may be more effective than those that target radical- or stroke-level handwriting.

### The role of semantics in character and sub-character handwriting

Our study shows that more concrete characters are associated with shorter character writing latencies, in line with findings that naming latencies are shorter for characters or words with higher concreteness (Liu et al., 2007). These results suggest that the concreteness of characters influences handwriting preparation, possibly by facilitating access to sensorimotor simulation (e.g., tactile imagery or visualization of the character’s meaning and its real-world referent) that supports the integration of semantic and word representations. Finally, compared with radical and stroke latencies, character latencies showed a greater decrease for characters with higher concreteness. This suggests that concreteness primarily facilitates early-stage lexical access (e.g., character retrieval), while downstream processes (e.g., radical or stroke retrieval) are less affected.

### The role of orthography in character and sub-character handwriting

We found that higher character frequency is associated with fewer occurrences of character amnesia, shorter character writing latencies, and shorter character writing durations. These findings align with previous research showing a facilitative effect of lexical frequency on handwriting in both alphabetic languages (e.g., Rapp et al., 2016; Rapp & Dufor, 2011) and Chinese (e.g., Wang et al., 2020; Zhang & Feng, 2017; Huang et al., 2021a, b). Similarly, characters acquired earlier in life are associated with lower rates of character amnesia and shorter writing preparation and execution times. More familiar context words are also associated with lower amnesia rates and shorter character writing latencies. These results are consistent with previous findings (Wang et al., 2020; Huang et al., 2021a, b), suggesting that characters that are more frequently encountered, learned earlier, or appear in familiar contexts have stronger, more stable representations in the orthographic lexicon, making them easier to retrieve and execute at the character level.

Additionally, our study found that these orthographic factors (e.g., character frequency and age of acquisition) facilitate writing preparation and execution at the radical or stroke levels. These findings are consistent with Lau (2020a, b), who also found that higher character frequency facilitates sub-character level writing latency. Our results show that character frequency modulates handwriting at the character and sub-character levels, underscoring the involvement of the orthographic lexicon within multilevel orthographic-motor integration (e.g., character, radical, and stroke levels). Importantly, these effects are larger at the character level than at the radical level, and larger at the radical level than at the stroke level, suggesting that the orthographic lexicon, which contains long-term orthographic representations, continues to influence handwriting at the sub-character level. However, its impact diminishes as orthographic representations are decomposed into smaller units (see next section for more discussion).

We also found that characters with fewer strokes are associated with lower character amnesia rates and shorter preparation and execution times. These results are consistent with previous research (Wang et al., 2020; Huang et al., 2021a, b) and challenge findings from small-sample studies suggesting that the number of strokes does not influence orthographic retrieval (Su & Samuels, 2010). Our findings imply that fewer strokes may reduce working memory load, allowing individuals to more efficiently program whole-word representations into graphemes (i.e., strokes) before and during handwriting execution. We also found that characters with a left-right or top-down composition facilitate character writing preparation and execution, suggesting that the visual-spatial aspect of radical programming influences character-level handwriting. This compositional effect may reflect habitual writing patterns, as writers are generally more accustomed to producing left-right structures or top-down composition than other configurations.

Additionally, the effects of stroke number and composition cascade to radical and stroke-level handwriting preparation and execution. Latencies and durations are more strongly influenced by stroke count and composition at the character level, followed by the radical level, and then the stroke level. These findings suggest that the graphemic buffer, a cognitive system that temporarily stores orthographic representations, is continuously engaged during character, radical, and stroke production but diminishes in influence as orthographic representations are decomposed into smaller units. Thus, character-level execution demands the highest orthographic working memory load in order to maintain the complete character representation. The radical level requires less memory load for processing smaller graphemic clusters. Strokes also serve as meaningful units that convey both linguistic and motor information to guide the execution of writing. These results further suggest that radicals may serve as orthographic chunks to reduce working memory load and as spatial templates to guide stroke execution (Yang et al., 2024). These results are consistent with previous studies demonstrating that participants had shorter handwriting latencies, fewer character amnesia instances when the prime and target shared the same phonetic radical (e.g., Xu et al., 2025b; Zhang & Wang, 2015; 呱 – 狐, “crying” – “fox”, sharing the phonetic radical 瓜 “melon”) or shared the same semantic radical (Xu et al., 2025b; Damian & Qu, 2019; semantic radical 米, meaning “rice”, in the character 糕, meaning “cake”) than when they did not share any radicals.

### Cascaded attenuation in handwriting

We observed that the effects of character frequency and age of acquisition cascade from character-level handwriting retrieval (i.e., character amnesia) and preparation (i.e., handwriting latency) to radical-level and stroke-level handwriting preparation. These lexical effects attenuate across different handwriting levels: they were strongest at the character level and weaker at the radical and stroke levels, with no significant difference between radical-level and stroke-level handwriting latencies. In addition, we observed that the effects of the number of strokes on handwriting preparation and execution cascade from characters to radicals and strokes, with weaker effects at the radicals and the weakest at the strokes (though note that it did not affect latencies at the stroke level). The findings suggest that lexical effects cascade from characters to strokes, gradually attenuating across levels. These cascaded and attenuated orthographic effects are consistent with previous findings showing that character frequency and the number of strokes modulate handwriting at both the character and sub-character levels, with the modulation being weaker at the latter than at the former level (Lau, 2020a, b). These findings suggest that during character-level handwriting preparation, orthographic representations are activated in long-term orthographic memory and decomposed into graphemes in working memory. The orthographic lexicon exerts a weaker influence on radical-level handwriting planning, likely because once a character has been selected from the orthographic lexicon, subsequent radical- and stroke-level planning relies less on lexicon-based representations and increasingly on temporarily maintained abstract orthographic information in the graphemic buffer. During handwriting execution, abstract orthographic representations are maintained in the graphemic buffer across character-level, radical-level, and stroke-level processing. However, the weaker influence of the graphemic buffer at the radical and stroke levels may reflect the fact that, as execution is decomposed into smaller units, it increasingly relies on automatic motor mechanisms (Lau, 2020a).

Our results also demonstrate that the effects of orthographic factors cascade and attenuate across all levels, while phonological and semantic factors are constrained by their hierarchical levels. Specifically, sub-character-level handwriting can rely on higher-level orthographic information (i.e., character-level variables such as character frequency and age of acquisition) from long-term memory. In contrast, sub-character-level handwriting cannot make use of higher-level phonological or semantic information. For instance, we observed that the radical-level variable, such as the effect of phonetic radical order on latency, cascaded from character-level handwriting to radical-level handwriting but not to stroke-level handwriting. Moreover, semantic effects (e.g., concreteness) observed at the character-level handwriting did not cascade to sub-character-level handwriting. These results suggest that phonological and semantic processes follow a level-hierarchy constraint under the cascaded attenuation hypothesis: character-level handwriting accesses both character-level variables (e.g., concreteness and homophones) and radical-level variables (e.g., phonetic radical order). However, radical-level handwriting only accesses radical-level variables, and stroke-level handwriting accesses only stroke-level variables. It is important to note that task demands can modulate the observed pattern of effects. Our handwriting-to-dictation task may emphasize the association between phonology and orthography, which may rely less on semantic activation than picture naming or character copying tasks. Therefore, most semantic factors did not influence handwriting, and the concreteness effect modulated character-level handwriting but did not cascade to sub-character-level handwriting, likely reflecting this task-specific constraint. Future research examining handwriting across different paradigms (e.g., character copying and picture-name handwriting) would help clarify how task demands shape engagement and the cascading of phonological, semantic, and orthographic systems.

We acknowledge that current analyses that aggregate radical- and stroke-level measures within each character do not capture fine-grained handwriting within each character or within-character variability, such as how the complexity of an earlier radical may modulate the latency or duration of a later radical. Future studies can employ more detailed radical- or stroke-level annotations and treat these units as nested observations within characters, which may provide a more precise characterization of how lexical variables shape radical- and stroke-level handwriting. Context-word familiarity ratings were obtained from 15 participants, as described by Wang et al. (2020). We acknowledge that this sample size is relatively small and may limit the generalizability of the familiarity norms.

**Fig. 10:**
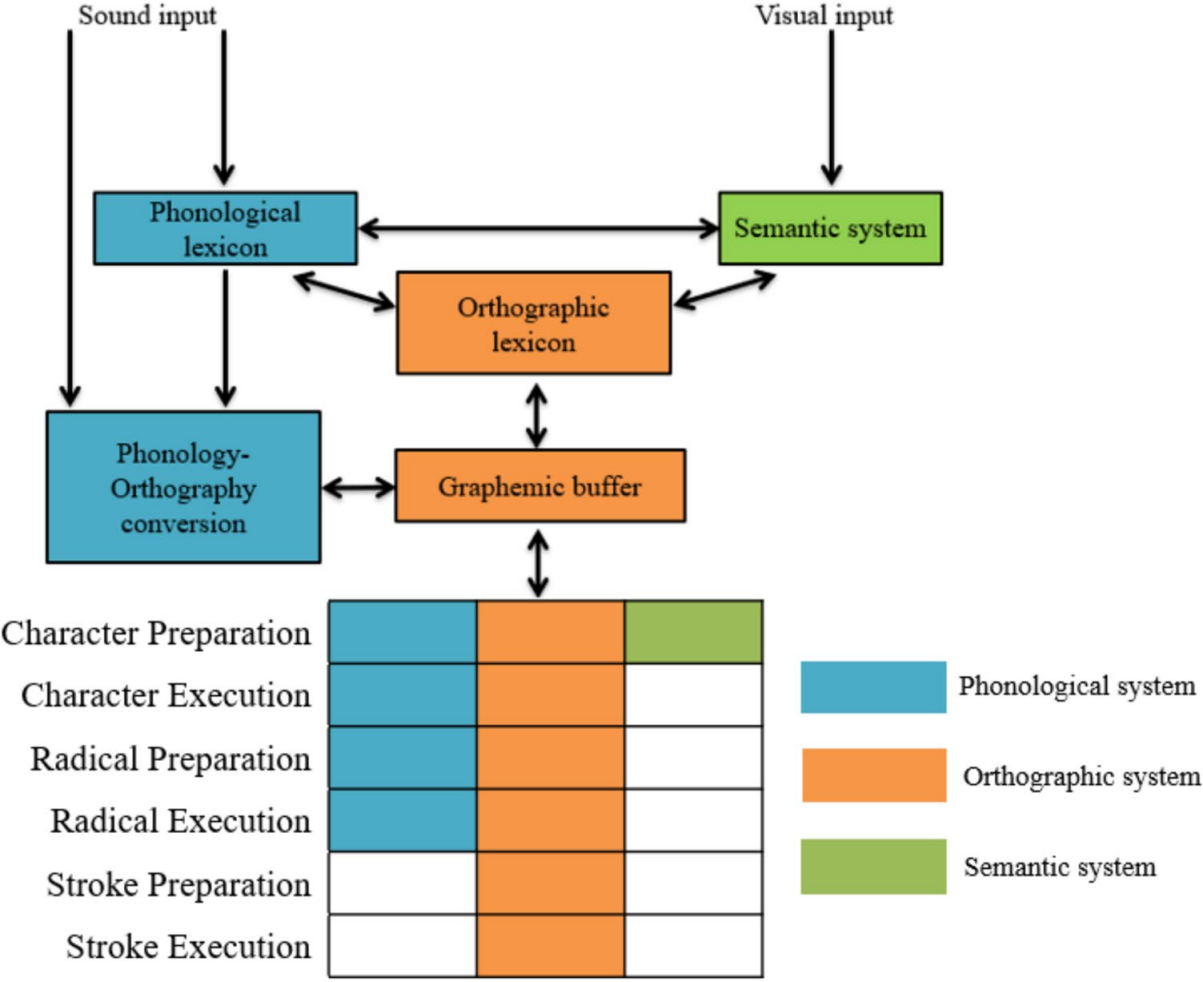
Updated cognitive model for handwriting at the character, radical, and stroke levels. We present a cascaded model of handwriting in which the phonological system modulates handwriting preparation and execution at the character and radical levels. The semantic system modulates handwriting preparation at the character-level. The orthographic system has an all-around effect

Collectively, these findings contribute to refining current handwriting models by highlighting the cascading effects of linguistic components across character, radical, and stroke-level writing preparation and execution (Fig. 10). When hearing a dictation prompt specifying a target character, handwriters first employ the phonological (e.g., the facilitative effects of phonetic radical order and character regularity on character writing latency; Wang et al., 2020) and semantic information (e.g., the more concrete meaning of the characters facilitates the access of the semantic representations (e.g., Liu et al., 2007) to guide access to the orthographic representations of characters in the orthographic lexicon. More stable orthographic representations (i.e., more frequent characters) are accessed more quickly from orthographic long-term memory during handwriting preparation and execution (e.g., facilitative effects of frequency on character writing latencies and durations; Rapp & Dufor, 2011; Rapp et al., 2016). The retrieved orthographic information is maintained in a graphemic buffer; characters with more strokes tend to be produced less accurately and/or more slowly (e.g., Wang et al., 2020; Huang et al., 2021a, b).

At the sub-character level of handwriting, the phonological system modulates writing preparation and execution via a different mechanism. Radical writing duration is shorter when the phonetic radical is written later in the sequence or when the character is not a phonogram. This pattern suggests that, once character-level planning is completed, handwriters rely more heavily on automated motor execution. When phonological information is either less involved (as in non-phonograms) or has already been processed earlier in the writing sequence, subsequent radical production is less susceptible to phonological interference, resulting in faster radical-level execution. Handwriters exhibit shorter radical writing latencies and shorter writing durations for characters with fewer strokes and more radicals. This pattern suggests that the graphemic buffer is less heavily loaded for characters with fewer strokes, and that radicals are retrieved as functional units rather than planned stroke by stroke. Such unit-based retrieval enables complex stroke patterns to be chunked into well-defined radical structures, thereby facilitating handwriting execution. In addition, faster handwriting for higher-frequency characters and characters with fewer strokes at the radical and stroke levels indicates that sub-character-level handwriting continues to rely on stable orthographic representations stored in the orthographic lexicon, while placing lower demands on orthographic working memory to facilitate handwriting.

### Possible applications of the handwriting database and OpenHandWrite_Toolbox

We expect a wide range of applications for the current handwriting database and toolbox. This database enables the development of rapid diagnostic tests for assessing orthographic retrieval abilities. In the digital age, reduced handwriting practice has led to the deterioration of explicit orthographic knowledge, resulting in widespread character amnesia. Despite this growing phenomenon, there is currently a lack of standardized diagnostic tests available to measure individuals’ ability in orthographic retrieval. To address this gap, Langsford et al. (2024) used this dataset to identify 30 characters that are most effective in discriminating adults’ character amnesia rates. Their findings demonstrated that the individual’s amnesia rate derived from these 30 items exhibited a strong correlation (*r* ≈ 0.90) with the amnesia rate obtained from a set of 1200 characters. These findings suggest that our database is useful for constructing a concise diagnostic test comprising 30 items to assess character amnesia in Chinese.

Recent studies have also employed penscripts (e.g., handwritten images) to evaluate individuals’ ability to produce characters with good penmanship (i.e., legible, aesthetically pleasing handwriting). For instance, the Children’s Handwriting Evaluation Scale (CHES; Phelps et al., 1985) relies on trained raters to assess the legibility of children’s penscripts based on criteria such as letter forms, slant, spacing, and overall appearance. In the Minnesota Handwriting Assessment (MHA; Reisman, 1999), children copied English words, while occupational therapists evaluated the penscripts on alignment, size, spacing, and form appearance (a similar approach is also taken in the Minnesota Handwriting Test, Reisman, 1993, and the Print Tool, Olsen & Knapton, 2006). However, these subjective assessment tools tend to be costly (e.g., requiring trained raters or teachers to evaluate handwriting) and are prone to individual bias. To address these issues, researchers can use the scripts in this database to develop an automated assessment system for penmanship. For instance, Xu et al. (2024) used penscripts from a large-scale traditional Chinese handwriting database, combined penmanship ratings for each handwritten sample, and trained a convolutional neural network to predict penmanship scores. This method achieved a remarkable performance with an overall normalized mean absolute percentage error of 9.82%, highlighting the potential of our database for automated systems of penmanship evaluation in different scripts.

OpenHandWrite_Toolbox provides a tool to investigate handwriting in the digital era. Handwriting literacy has largely declined due to the heavy reliance on digital devices (e.g., Almog, 2019). Empirical evidence has indicated that compared with handwriting practice, typing on a computer led to poorer language learning outcomes in terms of spelling (Cunningham & Stanovich, 1990; van Reybroeck & Michiels, 2018), letter recognition (Longcamp et al., 2005; Bara & Gentaz, 2011), and handwriting (Chen et al., 2016). Huang et al. (2021a) further demonstrated that college students with greater digital exposure (e.g., frequent smartphone or computer use) and less engagement with handwriting (e.g., time spent writing) were more likely to experience character amnesia. Our toolbox can be used to examine how digital typing impairs handwriting ability and whether this deterioration occurs primarily at the character, radical, or stroke level. This package can also be used to investigate the neurocognitive processes underlying Chinese character handwriting (see Xu et al., 2025a for more details).

Additionally, this package has the potential to pre-screen children for dysgraphia, a condition characterized by a specific difficulty in acquiring handwriting skills (Xu et al., 2026; Berninger et al., 2008; Gosse & Van Reybroeck, 2020; Kandel et al., 2017; McCloskey & Rapp, 2017). Research has shown that children with dysgraphia exhibit poorer performance in writing characters with lower frequency (e.g., Marinelli et al., 2017; Afonso et al., 2015; Reich et al., 2003), lower regularity (e.g., Brunsdon et al., 2005; Cholewa et al., 2010), or more stroke counts (e.g., Roncoli & Masterson, 2016; Yachini & Friedmann, 2010). Our package can capture the degree of variation in handwriting ability by analyzing character-, radical-, and stroke-level features across groups. This enables researchers to investigate the cognitive mechanisms underlying developmental dysgraphia and aid in the development of effective diagnostic tools for handwriting difficulties.

## Conclusion

This study introduces a large-scale database of Chinese character handwriting and demonstrates how lexical variables influence writing latencies and durations at the character, radical, and stroke levels. Our study highlights the cascading effects of linguistic components on all three levels of writing preparation and execution. This database was collected using the upgraded OpenHandWrite_Toolbox, a user-friendly, open-source toolbox. It includes a GUI for intuitive experiment design and batch-processing scripts to extract various handwriting measurements. The toolbox is designed to be used, adapted, and modified by a wide range of researchers for future handwriting studies.

## Supplementary Information

Below is the link to the electronic supplementary material.

Supplementary file1 (PDF 668 KB)

## Data Availability

All stimuli, data, and codes are available on the Open Science Framework (https://osf.io/rn2ck/?view_only=4e2fc80957314e53a2afd47a1db8f217).
