# Supplementary material for: A stroke-level large-scale database of Chinese character handwriting and the OpenHandWrite_Toolbox for handwriting research
Source: Behav Res Methods. 2026 May 11;58(6):162. doi: 10.3758/s13428-026-03001-4 (PMC13160981; doi:10.3758/s13428-026-03001-4)
Supplement: Supplementary file 1 — Supplementary file1 (PDF 668 KB) [file 13428_2026_3001_MOESM1_ESM.pdf]

Appendix A. Guide to experiment building

OpenHandWrite's GetWrite has been typically used with PsychoPy's scripting interface (https://github.com/isolver/OpenHandWrite/wiki/GetWrite-Experiment-Template). In this case, an experiment needs to be implemented in Python scripts by invoking PsychoPy and GetWrite's functionalities programmatically. While such a programming approach is the most flexible and powerful way to create experiments, it can be non-intuitive and demanding for researchers who are not experienced in Python. To ease the challenges in developing and running handwriting experiments, we provide a fully functional template experiment that can be opened and edited with PsychoPy Builder. A user should launch PsychoPy Builder by running the PsychoPyBuilder.bat file bundled with the OpenHandWrite distribution (downloadable at https://github.com/isolver/OpenHandWrite/releases), which provides built-in access to GetWrite. For a comprehensive introduction and tutorial to PsychoPy Builder, see Peirce and MacAskill (2018).

A conceptual flowchart of the critical procedures of our template experiment is illustrated in **Appendix Figure 1**. At the beginning of the experiment, the program initializes the tablet monitoring interface provided by GetWrite. Then, a pen position validation procedure is run, during which a black circle will appear at nine different locations on the screen, and the participant needs to use the tablet pen to press at a central white point inside the circle each time. After validation, the experiment enters the sequence of experimental trials (see **Apparatus and procedure** for details). Within each trial, the participant's pen movement data is recorded during the writing phase, and various trial variables (e.g., handwriting latency, duration, and self-reports) are saved. Finally, the tablet monitoring interface is closed at the end of the experiment.

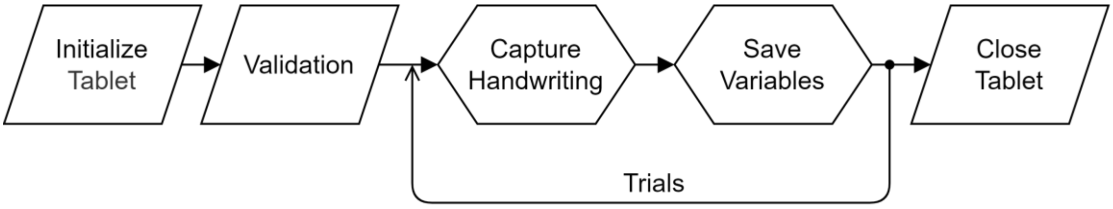

Appendix Figure 1. Critical procedures in the PsychoPy experiment.

A user familiar with the PsychoPy Builder GUI would find that most parts of our template are not different from a regular PsychoPy experiment. The overall *flow* of the experiment is constructed by combining various *routines*, which represent the steps of the experiment (e.g., showing instructions, recording handwriting data, collecting self-report responses). A routine consists of one or more *components*, which are the building blocks of an experiment and define the actual behaviour of the routine. For example, the self-report routine contains a text component for displaying the guide text, and a keyboard component for monitoring and collecting the participant's response. A series of routines can be executed repeatedly by grouping them into a *loop*, which is typically used for iterating experimental trials. Trial content (e.g., audio stimulus, target character) and condition information can be conveniently defined in a CSV (comma-separated values) file or Excel sheet (this is called the "condition file"). However, for handwriting experiments, there are a few special requirements regarding the format of the condition file (see **Saving trial variables** below). Because GetWrite is a programming library, there are no GUI components in PsychoPy Builder for accessing GetWrite's functionalities. As a solution, we used PsychoPy Builder's Code component to invoke GetWrite with custom inline codes. In the following sections, we detail the Code components in our template that are critical to capturing handwriting and saving trial variables.

Initializing and closing the tablet device

GetWrite uses PsychoPy's ioHub framework for parallel monitoring of pen movements. We use the Code component `init_io` in the `init_seq` routine to start the ioHub-based tablet device (the `tablet` object). Inside this component, the `start_iohub` function begins the ioHub service; the name of the data output file should be passed to the function's first argument. The `data.importConditions` method reads all trial information from the condition file (which is passed to the first argument of the method), and the `io.createTrialHandlerRecordTable` method associates the trial information with the ioHub service. At the end of the experiment, we use the `end_io_code` component in the `end_io` routine to stop the tablet device. At the very beginning of the experiment, we also initialised two visual objects, `pen_pos_stim` and `pen_traces_stim` (inside the `init_wintab` component of the `init` routine), for displaying the latest pen tip location and the existing handwriting traces.

Capturing handwriting

Capturing/recording of handwriting is started by setting the `tablet.reporting` property to `True` (`tablet.reporting = True`), and is paused by setting the property to `False` (`tablet.reporting = False`). During each trial, we start recording handwriting before the cue sound (the `main_aud_pen` component of the `main_audio` routine) and stop recording after the participant presses the space bar (the `main_pen` component of the `main_trial` routine). Because participants write characters on paper sheets with a digital ink pen tablet in our experiment, we chose not to draw handwriting traces on the screen to minimize distraction. However, pen traces can be easily shown by uncommenting the following Python code in `main_pen` (under the "Each Frame" tab of the component): `pen_traces_stim.updateFromEvents(pen_samples)`.

Saving trial variables

Different from typical PsychoPy experiments, trial variables are saved into an HDF5 file instead of a CSV file because data saving is handled by the ioHub service for GetWrite experiments. Each participant's trial variables, along with the recorded handwriting processes, will be saved to the HDF5 file under the "*data*" folder. In our experiment, the `save_vars_code` component in the `save_vars` routine is responsible for storing participant ID, self-report responses, and several auxiliary variables.

Condition file format and data saving

With the current version of OpenHandWrite (v0.4.9), there are three consequences of using the ioHub service for data saving. First, any variable that needs to be saved into the HDF5 file *must* appear in the "condition file". For example, if we want to record participant ID and self-report responses, we must define these variables in the condition file (see the two rightmost columns in **Appendix Figure 2**), even though their values are not determined before the actual experiment is done. Second, the placeholder values for these variables *must* match the data types of their expected resulting values. For example, because self-report responses are expected to be an integer number (0=correct, 1=character amnesia, 2=did not know the correct writing), we used a negative *integer* number (-1) as placeholders. We also used the *character* "x" as the placeholder for participant ID, whose value is expected to be a string of characters. Third, the variables *must* be assigned with their actual values during the experiment (e.g., the variable for self-report must be given the actual participant response codes), and they *must* be saved with the ioHub service (this is done by the `io.addTrialHandlerRecord` method in the `save_vars_code` component).

	A	B	C	D	E	F	G	H	I	J	K
1	DV_TRIAL_ID	DV_AUD_ONSET	DV_AUD_OFFSET	DV_TRIAL_START	DV_TRIAL_END	ROW_INDEX	audio_name	text	target	participant_id	self_report
2	-1	-1.1	-1.1	-1.1	-1.1	1	List1\1ba4.wav	罢了的罢	罢	x	-1
3	-1	-1.1	-1.1	-1.1	-1.1	2	List1\2ze2.wav	选择的择	择	x	-1
4	-1	-1.1	-1.1	-1.1	-1.1	3	List1\3diao1.wav	雕像的雕	雕	x	-1
5	-1	-1.1	-1.1	-1.1	-1.1	4	List1\4guo1.wav	黑锅的锅	锅	x	-1
6	-1	-1.1	-1.1	-1.1	-1.1	5	List1\5feng3.wav	讽刺的讽	讽	x	-1
7	-1	-1.1	-1.1	-1.1	-1.1	6	List1\6hai4.wav	害怕的害	害	x	-1
8	-1	-1.1	-1.1	-1.1	-1.1	7	List1\7fang1.wav	芳香的芳	芳	x	-1

Appendix Figure 2. An example condition file.

Auxiliary variables

Several important auxiliary variables *must* be defined in the condition file and their actual values also *must* be saved during the experiment. The variable `DV_TRIAL_ID` must correspond to the actual index of the trials (after possible randomization during the experiment). `DV_TRIAL_START` and `DV_TRIAL_END` must store the starting and ending timestamps for the writing phase of each trial; they are critical because MarkWrite will need to extract pen movements according to the timestamps saved in the two variables. Because `DV_TRIAL_START` and `DV_TRIAL_END` are expected to be real numbers (which have decimal places), we used a negative *real* number (-1.1) as their placeholders. Users can define any number of additional variables if needed. For example, we defined `DV_AUD_ONSET` and `DV_AUD_OFFSET` for the onset and offset times of the auditory stimulus; we also stored their actuals values with the `save_vars_code` component.

Appendix B. Handwriting data segmentation and feature extraction

The MarkWrite application (v0.4.9) can load the resulting HDF5 file (from GetWrite) that captured the handwriting data and visualise the handwriting data (the Markwrite interface is shown in **Figure 2)**. The detected handwriting data includes when the pen in the air is within a pen tablet's capture range (the distance between the pen-tip and PTH-651 tablet is within 3mm), or the pen-tip contact with the tablet to provide the time and coordinates for each pen sample data point. MarkWrite can automatically detect the stroke boundaries based on whether the pen-tip touched the tablet during handwriting. The stroke boundaries are defined by the

onset and offset of the stroke. Specifically, the boundary starts when the tablet first detects a pen-pressure sample from the pen-tip, and ends when the tablet registers zero pen-pressure. This auto-segmentation enables us to determine the feature boundaries at stroke, radical or character level. MarkWrite also allows users to manually create feature boundaries, that is, users can determine whether they want to include all the consecutive non-zero pen-pressure samples depending on their research questions (e.g., Chinese writers will sometimes have continuous strokes between two radicals so when the auto-segmentation function is not able to recognize there are actually two radicals here, users can choose the boundaries manually). Segments can be nested (e.g., a radical segment can have several 'child' segments for strokes), and they form a segment tree within a specific character. The users can export the pen sample data report plus segments report with the being labelled strokes, radicals or characters.

Pen sample report & Raw sample data report plus segs report

There are different types of reports that can be exported from the MarkWrite application. We will mainly discuss two export functions that were being used in our R scripts to generate stroke/radical level database. These reports include all the responses recorded by GetWrite, users can further program with GetWrite's Psychopy interface to define additional variables they want to record (e.g., item number, participant id or self-report). Pen data would be recorded when the pen-tip is close enough that the tablet can detect the pen in the air data (zero pen-pressure) or when the pen-tip touched the tablet (non-zero pen-pressure), including x and y-axis locations, pen-pressure, timestamps, segment labels (if they were additionally defined in MarkWrite by the researcher), and velocity in x or y coordinates.

Extracting handwriting metrics

A series of R functions were created for extracting handwriting metrics and are contained in a single R script batch_funcs.R. Inside the script, parallel processing of multiple sessions' data is enabled by R's future package. The calculation of the metrics is based on the Pen Sample Report and Raw Sample Data Report Plus Segs Report, which exposes various information for each pen sample, including the timestamp, x and y coordinates, and pen-pressure. The two Reports can be exported by MarkWrite from the PsychoPy experiment's resulting HDF5 file. Batch export of the Reports can be done with the script run_batchreportgen.bat (which internally calls another script, batchreportgen.py). As detailed below, for each written character in the current database, we generate handwriting metrics at three levels: (1) character, (2) radical (manually segmented), and (3) stroke (see also **Appendix Figure 3**).

At the whole character level, character writing latency (**char_rt**) is equal to duration from offset of the stimulus to the pen-tip firstly touched the tablet on this trial (onset of character handwriting). Character writing duration (**char_dur**) is the duration from the onset of pen-tip firstly touched the tablet on this trial to the last pen sample with non-zero pressure. Character length (**char_len**) is the sum of the lengths of all its strokes belonging to the character, it was calculated by the linear distance between consecutive pen sample points based on the x and y coordinates. The average pen-pressure of the character (**char_press_avg**) is the mean pressure of all stroke samples of the character.

For radical metrics, continuous strokes that straddle two or more radicals (if there are any) will be divided up according to the radical segmentation. Radical writing latency (**radical_rt_rel**) is equal to the duration between offset of the last radical to onset of the current radical. Radical writing duration (**radical_dur**) is the duration between onset of the current radical to the end of the current radical. Radical length (**radical_len**) is the sum of the lengths

of the strokes belonging to the radical. The distance to the previous radical (**radical_dist**) is equal to the linear distance between first point of the current radical and last point of previous radical. The average pen-pressure of the radical (**radical_press_avg**) is the mean pressure of the radical's pen samples.

Among these, stroke-level metrics are the most fundamental and serve as the basis for computing higher-level metrics. Stroke writing latency (**stroke_rt_rel**) is calculated by subtracting the last stroke's offset time from the timestamp of the current stroke writing onset time. Stroke writing duration (**stroke_dur**) is the difference between the timestamps of the last and first samples. Stroke length (**stroke_len**) is calculated by summing up the linear distance between consecutive pen sample points (based on their x and y coordinates), which is analogous to connecting the samples with straight lines. The raw length is converted to millimeters by dividing it with the tablet's lines per millimeter (lpmm) parameter. The lpmm value is the spatial resolution of a tablet. This value can usually be found in a tablet's specification sheet and can be calculated manually: draw vertical and horizontal lines that cross the whole surface of the tablet, export a pen sample report from MarkWrite and find the maximal y or x coordinates, divide them by physical height or width of the tablet (in millimeters) and the result is the lpmm value. The distance to the previous stroke (**stroke_dist**) is the linear distance between the current stroke's first sample and the previous stroke's last sample. The average pen-pressure of the stroke (**stroke_press_avg**) is the mean pressure of the pen samples in the stroke.

Subject	DV_TRIAL_ID	ROW_INDEX	self_report	target	char_dur	char_rt	char_len	char_press_avg	rad_label	rad_dur	rad_rt_rel	rad_len	rad_dist	rad_press_avg	stroke_label	stroke_dur	stroke_rt_rel	stroke_len	stroke_dist	stroke_press_avg
1	192	10	0	稻	2520	1369	42.46	16728	1	849	NA	17.89	NA	16680	3	135	72	6.9	1.195	16749
1	192	10	0	稻	2520	1369	42.46	16728	1	849	NA	17.89	NA	16680	4	81	97	2.645	4.29	14948
1	192	10	0	稻	2520	1369	42.46	16728	1	849	NA	17.89	NA	16680	5	75	71	2.065	1.625	15049
1	192	10	0	稻	2520	1369	42.46	16728	2	544	123	7.245	5.76	14685	6	106	123	3.53	5.76	18220
1	192	10	0	稻	2520	1369	42.46	16728	2	544	123	7.245	5.76	14685	7	60	88	0.9	1.525	12859
1	192	10	0	稻	2520	1369	42.46	16728	2	544	123	7.245	5.76	14685	8	66	78	0.915	1.07	9191
1	192	10	0	稻	2520	1369	42.46	16728	2	544	123	7.245	5.76	14685	9	67	79	1.9	1.555	16521
1	192	10	0	稻	2520	1369	42.46	16728	3	924	80	17.33	1.535	17815	10	188	80	4.045	1.535	17949
1	192	10	0	稻	2520	1369	42.46	16728	3	924	80	17.33	1.535	17815	11	165	111	5.215	4.38	21782
1	192	10	0	稻	2520	1369	42.46	16728	3	924	80	17.33	1.535	17815	12	53	110	1.13	4.165	12311
1	192	10	0	稻	2520	1369	42.46	16728	3	924	80	17.33	1.535	17815	13	211	86	6.94	1.095	16065

Appendix Figure 3. Handwriting metrics for an example trial. Subject: number of the subject; DV_TRIAL_ID: number of trial; ROW_INDEX; item index; self-report: participant's report on handwriting, 0, 1, and 2 represent correct handwriting, character amnesia, and incorrect handwriting; target: the to be written character; char_dur: character writing duration; char_rt: character writing latency; char_len: character length; char_press_avg: the average pen-pressure of the character; radical_label: radical index; rad_dur: radical writing duration; rad_rt_rel: radical writing latency; rad_len: radical length; rad_dist: the distance to the previous radical; rad_press_avg: the average pen-pressure of the radical; stroke_label: stroke index; stroke_dur: stroke writing duration; stroke_rt_rel: stroke writing latency; stroke_len: stroke length stroke_dist: the distance to the previous stroke; stroke_press_ave: the average pen-pressure of the stroke.

In addition to extracting the numeric writing metrics above, our R script also features rich visualisation functionalities. It can automatically generate the plot for each written character in the *plots\char output* folder (**Figure 3**) and generate detailed stroke-by-stroke plots in the *plots\by-stroke* folder (**Figure 4**). To batch process the results of multiple experimental sessions, one can first organize the Pen Sample Reports and Raw Sample Data Report Plus Segs Reports exported from the HDF5 files into a single folder, and then write a R script to invoke the process_all_reports function defined in the batch_funcs.R script. Our script batch_summarise_all.R is a complete example for batch processing.

Appendix C. Regression results on pen-pressure

Character pen-pressure. The results of the regression model (R^2 = .601) are presented in **Appendix Table 1**. Orthography modulates pen-pressure: pressure is greater for characters with earlier age of acquisition, fewer strokes, or left-right composition. Neither semantic nor phonological predictors significantly affect pen-pressure.

Radical pen-pressure. The results of the regression model (R^2 = .542) are presented in **Appendix Table 1**. Phonology influences radical pen-pressure: pressure is greater when the characters are not phonograms, or their first radical is phonetic radical. Semantic predictors also modulate pen-pressure, with higher pressure for more imageable characters. Orthographically, greater pen-pressure associates with characters that contain fewer strokes, have fewer radicals, have a left-right composition, or with a top-down composition.

Stroke pen-pressure. The results of the regression model (R^2 = .605) are presented in **Appendix Table 1**. Orthography influences pen-pressure: greater stroke pen-pressure is associated with characters that are acquired earlier, contain fewer strokes, have a left-right composition, lack a top-down composition, or appear in more familiar context words. Neither phonological nor semantic factors influence stroke pen-pressure.

Appendix Table 1 Results of character-level, radical-level, stroke-level regressions on pen-pressure.

	Character pen-pressure			Radical pen-pressure			Stroke pen-pressure		
Linear term	*β*	*t*	*p*	*β*	*t*	*p*	*β*	*t*	*p*
(Intercept)	11886.32	83.23	**< .001*** **	13258.88	112.01	**< .001*** **	10475.48	69.83	**< .001*** **
Phonogram	-20.77	-0.94	0.531	-80.10	-2.44	**0.034***	4.21	0.16	0.876
Phonetic radical order	-18.23	-1.00	0.531	90.48	3.16	**0.005*** *	-4.22	-0.20	0.876
Regularity	16.76	0.88	0.531	16.49	0.57	0.726	-3.50	-0.16	0.876
Homophone density	9.57	0.58	0.676	2.72	0.11	0.940	13.55	0.71	0.683
Number of meanings	10.22	0.55	0.676	8.21	0.29	0.902	14.08	0.65	0.683
Imageability	46.02	1.68	0.261	99.68	2.37	**0.036**	19.85	0.62	0.683
Concreteness	-37.81	-1.38	0.391	-60.08	-1.43	0.239	-44.19	-1.38	0.338
Frequency	50.73	2.17	0.106	2.69	0.08	0.940	52.06	1.90	0.135
Age of acquisition	-89.17	-4.24	**< .001*** **	-53.82	-1.69	0.162	-58.90	-2.39	**0.048***
Number of strokes	-710.69	-21.66	**< .001*** **	-594.81	-16.61	**< .001*** **	-486.27	-17.55	**< .001*** **
Number of radicals	-8.07	-0.38	0.745	-341.88	-8.05	**< .001*** **	21.75	0.86	0.683
Left-right	120.88	4.92	**< .001*** **	413.07	10.27	**< .001*** **	194.62	6.73	**< .001*** **
Top-down	-7.51	-0.33	0.745	-206.60	-5.18	**< .001*** **	-98.85	-3.54	**0.002*** *
Word familiarity	20.95	1.05	0.531	26.81	0.86	0.544	66.53	2.85	**0.016***
Length	16.32	4.67	**< .001*** **	-6.76	-1.20	0.232	337.76	17.64	**< .001*** **
Distance	16.32	4.67	**< .001*** **	-77.83	-3.55	**< .001*** **	-45.74	-1.11	0.267

Results of character-level regressions on character pen-pressure.
Significant p-values indicated in bold.
Key: β = coefficient, t = t-value, p = p-value

Hierarchical linguistic effects at different levels of writing pen-pressure: Radical pen-pressure is the largest, followed by character pen-pressure, and then stroke pen-pressure. Compared with character pen-pressure, radical pen-pressure has a larger increase for characters with a phonetic radical to be written first, with fewer radicals, being a left-right composition. Compared with stroke pen-pressure, character pen-pressure has a greater decrease for characters that contain more strokes. Compared with stroke pen-pressure, radical pen-pressure has a larger increase for characters with fewer radicals, being a left-right composition. (**Appendix Table 2** and **Appendix Figure 5**).

Appendix Table 2 Results of character-, radical-, and stroke-level regressions on pen-pressure.

	Level: Character vs. Radical			Level: Character vs. Stroke			Level: Radical vs. Stroke		
Linear term	*β*	*t*	*p*	*β*	*t*	*p*	*β*	*t*	*p*
(Intercept)	12439.0	124.70	**< .001*** **	11118.7	122.75	**< .001*** **	11677.5	165.92	**< .001*** **
Level	-1149.8	-5.76	**< .001*** **	1535.23	8.48	**< .001*** **	2672.88	18.99	**< .001*** **
Phonogram	-48.55	-2.45	**0.029***	-8.04	-0.47	0.635	-36.12	-1.73	0.118
Phonetic radical order	26.36	1.58	0.176	-10.58	-0.76	0.572	34.07	1.94	0.093
Regularity	14.12	0.82	0.484	7.03	0.48	0.635	4.42	0.24	0.809
Homophone density	5.80	0.39	0.699	11.37	0.90	0.523	7.60	0.48	0.681
Number of meanings	8.96	0.53	0.642	12.64	0.89	0.523	11.35	0.64	0.612
Imageability	73.82	2.95	**0.007** **	33.05	1.57	0.206	60.96	2.31	**0.042***
Concreteness	-46.38	-1.86	0.111	-40.90	-1.94	0.105	-49.45	-1.87	0.095
Frequency	25.79	1.21	0.289	51.22	2.85	**0.011***	26.29	1.17	0.310
Age of acquisition	-74.27	-3.86	**< .001*** **	-74.44	-4.59	**< .001*** **	-59.54	-2.93	**0.010***
Number of strokes	-660.27	-25.62	**< .001*** **	-595.70	-27.18	**< .001*** **	-546.64	-24.40	**< .001*** **
Number of radicals	-167.63	-7.42	**< .001*** **	9.69	0.59	0.635	-150.45	-6.30	**< .001*** **
Left-right	269.98	11.64	**< .001*** **	158.62	8.36	**< .001*** **	307.55	12.53	**< .001*** **
Top-down	-126.99	-5.81	**< .001*** **	-55.05	-3.06	**0.008** **	-174.66	-7.47	**< .001*** **
Word familiarity	24.01	1.31	0.266	43.45	2.83	**0.011***	46.57	2.40	**0.038***
length	3.92	1.20	0.231	177.97	19.64	**< .001*** **	165.57	14.66	**< .001*** **
Level×Phonogram	56.54	1.43	0.358	-25.45	-0.75	0.819	-81.39	-1.94	0.121
Level×Phonetic radical order	-89.52	-2.69	**0.025***	-15.28	-0.55	0.819	74.09	2.11	0.099
Level×Regularity	5.18	0.15	0.945	19.45	0.66	0.819	14.21	0.39	0.907
Level×Homophone density	7.50	0.25	0.945	-3.61	-0.14	0.955	-11.10	-0.35	0.907
Level×Number of meanings	2.33	0.07	0.945	-4.84	-0.17	0.955	-7.12	-0.20	0.907
Level×Imageability	-56.32	-1.13	0.455	25.93	0.61	0.819	82.05	1.55	0.242
Level×Concreteness	17.73	0.36	0.945	6.19	0.15	0.955	-11.58	-0.22	0.907
Level×Frequency	49.41	1.16	0.455	-1.01	-0.03	0.978	-50.42	-1.12	0.462
Level×Age of acquisition	-30.03	-0.78	0.677	-29.42	-0.91	0.819	0.56	0.01	0.989
Level×Number of strokes	-85.64	-1.66	0.271	-229.68	-5.24	**< .001*** **	-141.64	-3.16	**0.007** **
Level×Number of radicals	319.65	7.08	**< .001*** **	-35.52	-1.09	0.773	-354.01	-7.42	**< .001*** **
Level×Left-right	-299.49	-6.46	**< .001*** **	-75.54	-1.99	0.217	224.34	4.57	**< .001*** **
Level×Top-down	238.93	5.46	**< .001*** **	95.09	2.64	0.059	-143.60	-3.07	**0.008** **
Level×Word familiarity	-6.02	-0.16	0.945	-45.01	-1.47	0.499	-39.11	-1.01	0.487
Level× length	24.81	3.80	**< .001*** **	-323.30	-17.83	**< .001*** **	-348.11	-15.41	**< .001*** **

Significant p-values indicated in bold.
Key: β = coefficient, t = t-value, p = p-value.

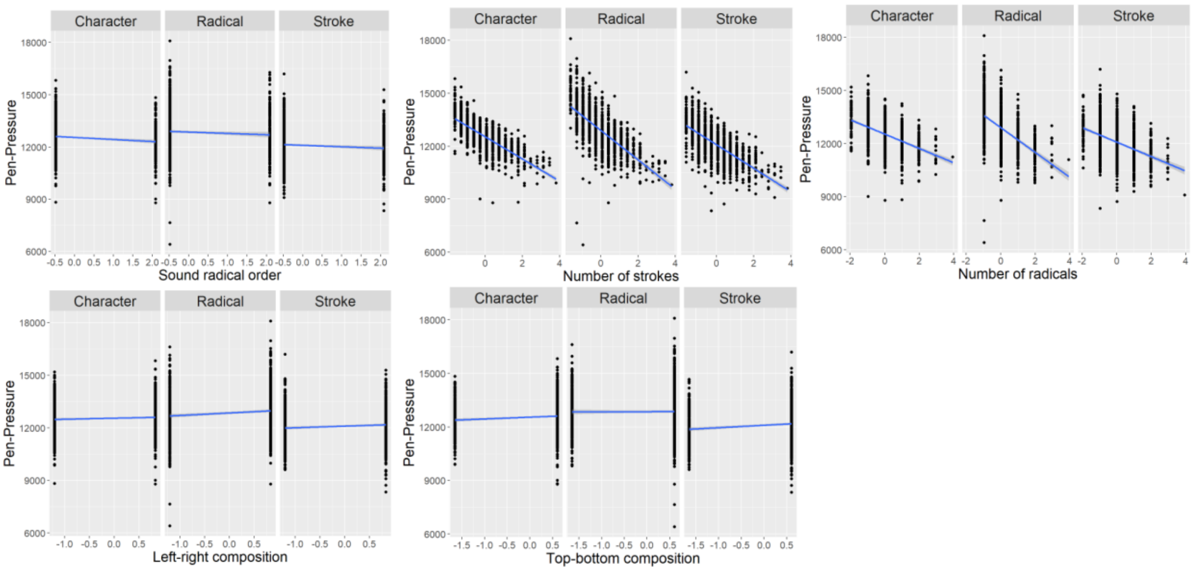

Appendix Figure 5. Significant interaction effects of pen-pressure as a function of lexical variables, as described in **Appendix Table 2**. The lexical variables were transformed into z-scores, include phonetic radical order, number of strokes, number of radicals, left-right composition, and top-down composition.
